# *Bordetella* adenylate cyclase toxin elicits chromatin remodeling and transcriptional reprogramming that blocks differentiation of monocytes into macrophages

**DOI:** 10.1128/mbio.00138-25

**Published:** 2025-03-19

**Authors:** Jawid Nazir Ahmad, Martin Modrak, Marketa Fajfrova, Blanca Martin-Borja Sotoca, Oldrich Benada, Peter Sebo

**Affiliations:** 1 Institute of Microbiology of Czech Academy of Sciences, Prague, Czechia; Fondazione Biotecnopolo di Siena, Siena, Italy

**Keywords:** macrophages, differentiation, monocytes, RTX toxins, cyclic AMP, *Bordetella pertussis*, epigenetics

## Abstract

*Bordetella pertussis* infects human upper airways and deploys an array of immunosuppressive virulence factors, among which the adenylate cyclase toxin (CyaA) plays a prominent role in disarming host phagocytes. CyaA binds the complement receptor-3 (CR3 aka α_M_β_2_ integrin CD11b/CD18 or Mac-1) of myeloid cells and delivers into their cytosol an adenylyl cyclase enzyme that hijacks cellular signaling through unregulated conversion of cytosolic ATP to cAMP. We found that the action of as little CyaA as 22 pM (4 ng/mL) blocks macrophage colony-stimulating factor (M-CSF)-driven transition of migratory human CD14^+^ monocytes into macrophages. Global transcriptional profiling (RNAseq) revealed that exposure of monocytes to 22 pM CyaA for 40 hours in culture with 20 ng/mL of M-CSF led to upregulation of genes that exert negative control of monocyte to macrophage differentiation (e.g., *SERPINB2, DLL1,* and *CSNK1E*). The sustained CyaA action yielded downregulation of numerous genes involved in processes crucial for host defense, such as myeloid cell differentiation, chemotaxis of inflammatory cells, antigen presentation, phagocytosis, and bactericidal activities. CyaA-elicited signaling also promoted deacetylation and trimethylation of lysines 9 and 27 of histone 3 (H3K9me3 and H3K27me3) and triggered the formation of transcriptionally repressive heterochromatin patches in the nuclei of CyaA-exposed monocytes. These effects were partly reversed by the G9a methyltransferase inhibitor UNC 0631 and by the pleiotropic HDAC inhibitor Trichostatin-A, revealing that CyaA-elicited epigenetic alterations mediate transcriptional reprogramming of monocytes and play a role in CyaA-triggered block of monocyte differentiation into bactericidal macrophage cells.

**IMPORTANCE:**

To proliferate on host airway mucosa and evade elimination by patrolling sentinel cells, the whooping cough agent *Bordetella pertussis* produces a potently immunosubversive adenylate cyclase toxin (CyaA) that blocks opsonophagocytic killing of bacteria by phagocytes like neutrophils and macrophages. Indeed, chemotactic migration of CD14^+^ monocytes to the infection site and their transition into bactericidal macrophages, thus replenishing the exhausted mucosa-patrolling macrophages, represents one of the key mechanisms of innate immune defense to infection. We show that the cAMP signaling action of CyaA already at a very low toxin concentration triggers massive transcriptional reprogramming of monocytes that is accompanied by chromatin remodeling and epigenetic histone modifications, which block the transition of migratory monocytes into bactericidal macrophage cells. This reveals a novel layer of toxin action-mediated hijacking of functional differentiation of innate immune cells for the sake of mucosal pathogen proliferation and transmission to new hosts.

## INTRODUCTION

The pertussis agent *Bordetella pertussis* is a Gram-negative aerobic coccobacillus that infects the upper airways of humans and eventually can provoke fatal pneumonia in infants and the elderly (1–3). To proliferate on ciliated airway epithelia, the bacteria deploy an array of functionally redundant adhesins, several complement resistance factors, and at least four immunomodulatory protein toxins, including the notoriously known pertussis toxin playing a key role in pertussis pathology by eliciting the characteristic hyperleukocytosis (4, 5). The other potent immunosubversive toxin of classical *Bordetella* is the cell-invasive adenylate cyclase toxin-hemolysin (CyaA, ACT, AC-Hly) that plays a key role in subversion of both the innate and adaptive immune responses of host mucosa to *B. pertussis* infection (6–8). Binding to the CD11b subunit of the complement receptor 3 (CR3, aka the CD11b/CD18 α_M_β_2_ integrin, or Mac-1), the CyaA toxin disarms the bactericidal functions of host phagocytes by delivering into their cytosol its N-terminal adenylyl cyclase (AC) enzyme domain (9). This is activated by calmodulin and catalyzes the uncontrolled conversion of cytosolic ATP to the key second messenger signaling molecule, cAMP. The signaling of CyaA-produced cAMP through protein kinase A (PKA) and the Exchange protein directly activated by cAMP (Epac) then deregulates numerous cellular signaling pathways and instantly ablates the bactericidal capacities of sentinel phagocytes, such as oxidative burst and neutrophil extracellular trap formation (10, 11). CyaA action further blocks opsonophagocytic uptake and killing of bacteria by macrophages (12) and eventually provokes their apoptosis through activation of the Bim-Bax pro-apoptotic cascade (13).

Circulating monocytes (CD14^high^, CD11b^+^) play an important role in host defense and are chemoattracted into infected or injured tissue where they differentiate into a range of effector cells, such as dendritic cells, macrophages, or bone-remodeling osteoclasts (14). Recently, we have shown that cAMP signaling of as low CyaA concentrations as 22 pM (4 ng/mL) through hijacking of PKA provokes an arrest of M-CSF-elicited *ex vivo* differentiation of primary human circulating monocytes into bactericidal macrophage cells. Moreover, CyaA/cAMP signaling could trigger *ex vivo* de-differentiation of terminally differentiated primary human alveolar macrophages back into a monocyte-like cell type, whereas treatment with equal amounts of the enzymatically inactive CyaA-AC^−^ toxoid, unable to convert ATP into cAMP, had no impact (15, 16). Therefore, in this study, we analyzed the transcriptional reprogramming of primary human monocytes exposed to the action of such a low CyaA toxin concentration. We show that sustained cAMP signaling elicited by low CyaA amounts triggers epigenetic modifications that are accompanied by enhanced accumulation of transcriptionally repressive heterochromatin in monocyte nuclei and that the CyaA toxin activity provokes alteration of transcription of numerous genes involved in immune functions, upregulating genes that interfere with monocyte to macrophage differentiation.

## RESULTS

### CyaA/cAMP signaling deregulates gene expression involved in monocyte differentiation

**Fig 1:**
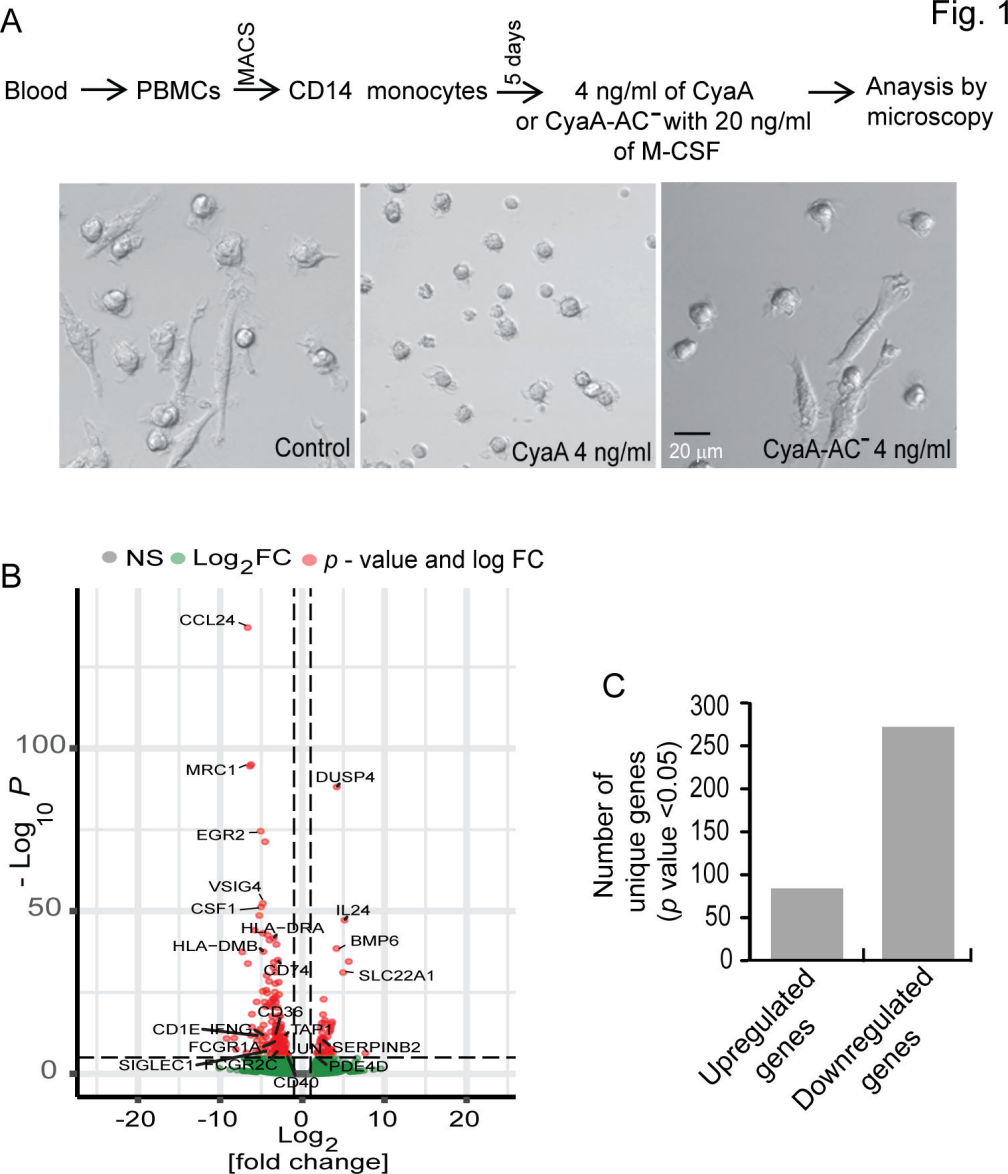
CyaA-elicited arrest of monocyte differentiation is accompanied by transcriptional reprogramming. (**A**) Scheme of the experimental procedure for analysis of differentiation of monocytes. CD14^+^ monocytes were exposed either to 4 ng/mL of CyaA, or to catalytically inactive toxoid CyaA-AC^−^, or were mock-treated in the presence of recombinant human M-CSF (20 ng/mL), and the cells were analyzed 5 days after exposure to toxin. Representative bright field images are shown. (**B**) Volcano plot of the DEGs after culture of CD14^+^ monocytes in DMEM containing 20 ng/mL M-CSF and 4 ng/mL CyaA, compared to gene expression levels in mock-treated control cells. Gene expression alterations are given as fold change (FC) *vs P*-value. Spots corresponding to genes with *P* ≤ 0.05 and log_2_ FC ≥ 1 are colored in red, and spots representing genes with *P* ≥ 0.05 and log_2_ FC ≤ 1 (not significant) are colored in green. Spots of genes with the highest fold change are annotated. (**C**) Total number of significantly altered genes (*P* ≤ 0.05 and log_2_ FC ≥ 1) in CD14^+^ monocytes after culturing in DMEM containing 20 ng/mL of M-CSF with or without 4 ng/mL of CyaA.

We have previously demonstrated that cAMP signaling resulting from exposure to very low concentrations of the CyaA toxin (22 pM) blocks the differentiation of migratory CD14^+^ monocytes into bactericidal macrophages (15). The circulating CD14^+^ monocytes cultured *ex vivo* for 5 days with 20 ng/mL M-CSF and 4 ng/mL of CyaA (22 pM) remained small, rounded, and undifferentiated (Fig. 1A). In contrast, M-CSF-stimulated control cells cultured in the absence of CyaA, or in the presence of the enzymatically inactive CyaA-AC^−^ toxoid unable to convert cellular ATP to cAMP (17), remained adherent and differentiated into large macrophage cells (Fig. 1A). To analyze the pathways involved in the block of monocyte differentiation, we performed unbiased deep sequencing of the transcriptome (RNAseq) of toxin-exposed cells. Highly purified circulating human CD14^+^ monocytes (>95% purity, Fig. S1) of anonymous blood donors were sub-cultured *ex vivo* for 40 hours in triplicates with the differentiating stimulus of human M-CSF (20 ng/mL) and in the presence or absence of 4 ng/mL (22 pM) of CyaA. The alterations of global gene expression profiles were next analyzed by RNAseq technology using total cellular RNA that was reverse transcribed into cDNA and analyzed by next-generation sequencing. Differentially expressed genes (DEG) in the control and toxin-treated cell samples were identified using a cutoff of >2-fold change at a *P* value < 0.05. This yielded 356 genes with altered transcription (listed in Table S1) depicted in the volcano plot with annotated spots of genes with the highest fold change (Fig. 1B), with 85 upregulated and 271 down-regulated genes (Fig. 1C).

Out of the genes with significantly altered transcription levels, we further focused on those relevant for host-pathogen interaction, monocyte-macrophage transition, and for which published findings allow to formulate interpretable hypotheses on the impact of the alteration of transcription. The genes with most prominently altered transcription levels (Fig. 1B) included genes involved in innate and adaptive immunity, such as *CCL24* encoding for an eosinophil attracting chemokine, *MRC1* a macrophage marker gene*, EGR2* a zinc finger transcription factor gene, *CSF1* encoding a growth factor for monocyte differentiation to macrophages, the *HLA-DRA* and *HLA-DMB* genes for molecules involved in antigenic peptide presentation on cell surface to T cells, *VSIG4* a macrophage signature gene (18)*, DUSP4* phosphatase that inactivates MAP kinases (19), zinc importer *SLC39A14,* which imports zinc ions required for the activation of some HDACs and the iron homeostasis regulating BMP6 gene.

**Fig 2:**
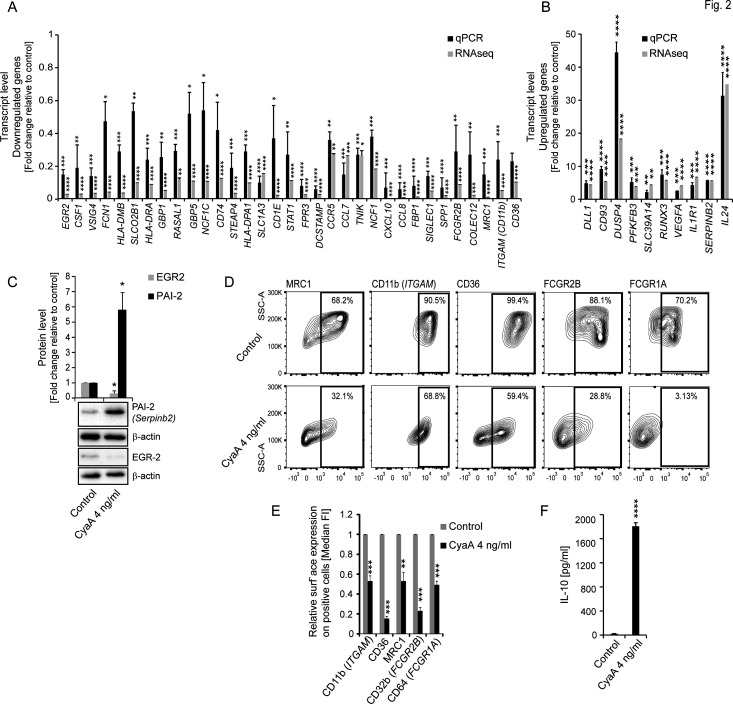
Validation of RNAseq data by qPCR and protein product detection. (**A, B**) Total RNA was extracted from CD14^+^ monocytes after 40 hours of incubation with CyaA (4 ng/mL), reverse-transcribed, and the mRNA transcripts of indicated genes were quantified by qPCR using appropriate primers (Table S2). Alteration of transcription is shown as fold change relative to the transcript levels in control cells after normalization to β-actin and β-2 microglobulin mRNA levels. *P < 0.05, **P < 0.005; ***P < 0.0005, ****P < 0.00001. (**C**) After 40 hours of exposure to CyaA (4 ng/mL), the CD14^+^ monocytes were lysed and protein levels were detected by specific antibodies using immunoblot. ImageJ software was utilized to quantify the protein levels that were normalized to the β-actin band intensity and are shown as fold change relative to the expression in mock-treated cells, *n* = 4, *P < 0.05. (**D**) CD14^+^ monocytes were cultured with CyaA (4 ng/mL) and 20 ng/mL M-CSF for 5 days and cells were analyzed for surface marker levels by FACS using directly labeled specific antibodies and isotype controls, *n* = 4. Percent of cell population expressing the indicated protein is shown by representative contour plots. (**E**) Decreased relative surface expression of indicated markers on positive cells is shown as fold change relative to control (median fluorescence intensity [MFI]), *n*  = 4; **P < 0.005; ***P < 0.0005. (**F**) CD14^+^ monocytes were treated with CyaA toxin (4 ng/mL, 22 pM) or mock-treated for 40 hours. The levels of IL-10 secreted into the media were quantified using ELISA (*n* = 3, ****P < 0.00001).

To verify that the RNAseq results can be generalized, we isolated monocytes from the blood of multiple anonymous healthy donors, treated them with 22 pM CyaA for 40 hours in the presence of 20 ng/mL of M-CSF, and quantified the transcription levels of a set of genes by qPCR using specific primers (Table S2). These results provided a robust validation of the transcriptional trends observed by the RNAseq analysis of triplicate samples of monocytes from a single donor (Fig. 2A and B). The qPCR analysis on cells from multiple donors confirmed that the selected genes were deregulated by the cAMP elevating capacity of the CyaA toxin (Fig. S2), with the caveat that qPCR quantification could not be used for validation of CyaA-triggered alterations of transcripts with a copy number of 100 per sample, or less. As further verified for selected genes by determination of protein product levels using immunoblotting, the *EGR2* and *SERPINB2* encoded proteins EGR2 and PAI-2 amounts followed the trends of CyaA-elicited transcript level alterations (Fig. 2C). Next, we validated the RNAseq data by FACS analysis, confirming the CyaA action-triggered reduction of cell surface levels of the protein products of five genes (e.g., *MRC1, ITGAM, CD36, FCGR2B* and *FCGR1A*) that encode for the Fc receptors FcγR1A and FcγR2B, and the macrophage markers CD36, CD11b, and MRC1 (Fig. 2D and E). Furthermore, the determination of the levels of secreted IL-10 in the culture media of CyaA-exposed monocytes revealed a significant increase in IL-10 secretion, corroborating the finding of the RNAseq analysis that CyaA action elicits the overproduction of IL-10 (Fig. 2F). All these performed assays, thus, validated the RNAseq results for further analysis.

### Exposure of CD14^+^ monocytes to CyaA toxin triggers inhibition of immune gene expression and transcription of genes required for differentiation into macrophages

**Fig 3:**
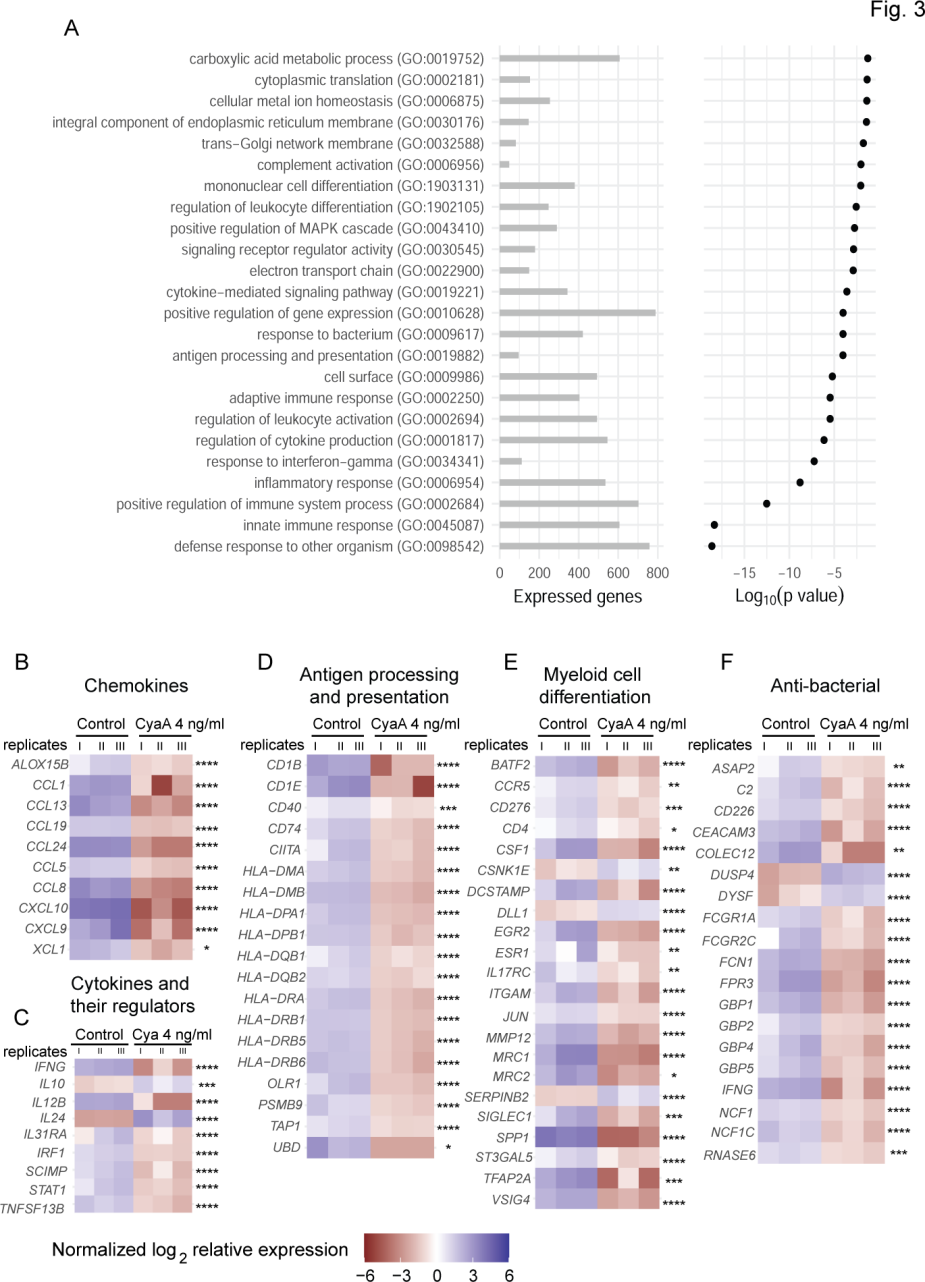
Clustering of functional categories of genes with altered transcription. (**A**) Selected gene ontology (GO) terms associated with genes exhibiting altered transcription levels after 40 hours of monocyte exposure to 22 pM CyaA toxin. Enrichment was assessed based on the ranking of genes by fold change using clusterProfiler (20). Horizontal axis shows the number of genes for each GO term that were at least minimally expressed in our data set (at least 60 reads in total over all samples), where the same gene can be annotated with multiple GO terms. (**B and C**) Chemokine and cytokine, (**D**) antigen processing and presentation, (**E**) myeloid cell differentiation, and (**F**) anti-bacterial pathways log_2_ ratios of absolute expression values. Induced genes are represented in purple and repressed genes are rendered in maroon color. *n* = 3, *P < 0.05, **P < 0.005, ***P < 0.0005, ****P < 0.00001.

To corroborate the analysis of transcriptional alterations elicited by CyaA action, gene set enrichment analysis of RNAseq data was performed using clusterProfiler (20). Among the enriched terms were primarily those representing host “defense response to organism,” e.g., primarily the innate immune response to infection, regulation of the immune system, and multiple terms related to the cytokine and chemokine secretion. Additionally, many terms were related to inflammatory response (Fig. 3A). Furthermore, terms related to antigen processing and presentation, leukocyte differentiation, complement activation, metabolic processes, regulation of MAPK cascade, and cellular ion homeostasis were quite prominent. The genes that are known to be involved in host immune response to infection and in monocyte differentiation were next selected for further analysis, listing the selected downregulated genes in key functional categories in Table 1 and the upregulated genes in Table 2. The genes with the most altered levels of transcription were then grouped by cellular processes for visualization of transcription level alterations for individual genes. As shown in Fig. 3B through F, CyaA action led to important downregulation of transcription of many genes essential for recruitment of circulating immune cells, antigen presentation, myeloid cell differentiation, and bactericidal functions. The CyaA-treated CD14^+^ monocytes exhibited reduced expression of genes for chemokines that drive migration of immune cells, such as *CXCL9, CXCL10, CCL8,* and *CCL24* (Fig. 3B) and cytokines that regulate the key bactericidal functions of myeloid cells, such as IFNγ and IL12B (Fig. 3C). Indeed, IFNγ levels are known to trigger the production of chemokines like *CXCL9* and *CXCL10* (21). CyaA action triggered concurring downregulation of genes involved in antigen processing, loading, and presentation to T cells, such as the *CIITA, HLA-DRA, HLA-DMA, HLA-DMB, CD74, TAP1, CD1B*, *PSMB9,* and *OLR1* (Fig. 3D). Hence, CyaA exposure locked the CD14^+^ monocytes in a state of reduced capacity to present peptide antigens through the MHC class I and class II pathways to trigger adaptive T cell immune responses.

**TABLE 1:** Set of downregulated genes in key functional categories

Regulators	Functions/processes
*CXCL10, CCL24, CCL8, CXCL9, CCL13, CCL1, CCL19, CCL5, ALOX15B, XCL1*	Chemotaxis
*IL12B, IFNG, TNFS13B, SCIMP, IRF1, STAT1, IL31RA*	Cytokines and their regulators
*UBD, CD1B, CD1E, HLA-DMB, HLA-DPA1, HLA-DRB6, HLA-DQB1, HLA-DRA, HLA-DRB5, HLA-DMA, HLA-DPB1, OLR1, CIITA, HLA-DRB1, CD74, HLA-DBQ2, PSMB9, TAP1, CD40*	Antigen processing and presentation
*SPP1, TFAP2A, EGR2, CSF1, VSIG4, MMP12, SIGLEC1, ITGAM, BATF2, MRC2, ST3GAL5, CD276, IL17RC, CD4, CCR5, ESR1, CD36, DC-STAMP, MRC1, JUN*	Myeloid cell differentiation
*FBP1, APOL4, CDS1, ME1, CD36, SLC1A3, HK3, APOL6, APOL1, APOL3*	Cell metabolism
*COLEC12, FPR3, FCN1, GBP1, IFNG, CEACAM3, GBP4, FCGR1A, FCGR2C, C2, RNASE6, ASAP2, NCF1C, NCF, GBP2, GBP5, CD226*	Phagocytosis/anti-bacterial
STEAP4, SLCO2B1	Metal ion and heme importer
SLC9A7, SYNPO2, CTTN	Lamellipodia formation, ER, and Golgi size regulation

**TABLE 2:** Upregulated genes in key categories

Regulators	Functions/processes
*IL24, IL10*	Block of Th17 differentiation, Th1 dysfunction, NFκB inhibition
*SERPINB2, DLL1, CSNK1E*	Negative regulators of monocyte to macrophage differentiation
*DYSF*	Inhibits phagocytosis
*SLC25A37, SLC39A14, BMP6*	Metal ion homeostasis
*KANK2*	Block of gene expression *via* SRC proteins
*TNFRSF21*	Pro-apoptotic, triggers Bax oligomerization

Most importantly, transcription of a whole set of genes involved in myeloid cell differentiation and monocyte to macrophage transition was strongly downregulated, providing a mechanistic explanation for the observed CyaA-elicited block of monocyte to macrophage differentiation process (15). Among the most strongly downregulated genes was the *CSF1* gene encoding for a growth factor that directs monocyte differentiation to macrophages by autocrine signaling. Transcription of the *EGR2* gene for a transcription factor regulating the monocyte differentiation to macrophages (22, 23) was prominently downregulated, as well as the *JUN* gene for the AP-1 transcription factor that initiates the nuclear signaling of M-CSF and, thus, drives monocyte differentiation to macrophages (24). Moreover, transcription of a set of macrophage-specific signature genes was importantly suppressed, such as of the *ITGAM*, *MRC1*, *VSIG4, SIGLEC1,* and *CD36* genes, respectively (Fig. 3E).

In turn, CyaA/cAMP signaling action triggered upregulation of genes for inhibitors of myeloid cell differentiation (Table 2 and Fig. 3E), such as the *SERPINB2* gene for plasminogen activator inhibitor 2 (PAI-2) that impairs monocyte differentiation and the genes *CSNK1E* for Casein Kinase 1 Epsilon, the activities of which are known to interfere with myeloid cell differentiation and promote rounded cell appearance (Fig. 3E) (25, 26). Finally, CyaA action further upregulated transcription of the *IL10* gene for the immunosuppressive cytokine IL-10 that downregulates the immune response to pathogens (Table 2 and Fig. 3C) (27).

In the category of genes directly involved in crucial antibacterial defense mechanisms, such as phagocytic uptake and killing of bacteria opsonized by complement components and/or by antibodies, there was downregulation of transcription of genes for the Fc receptor family, such as *FCGR1A,* and the *ASAP2* gene encoding a protein located beneath the phagocytic cup of the IgG-opsonized particle (28). Moreover, toxin action not only suppressed transcription of genes encoding crucial components of the phagocytosis machinery but also upregulated genes such as *DYSF* for the dysferlin protein that blocks phagocytosis (29). On the side of complement-mediated phagocytosis, CyaA action reduced transcription of genes encoding the C3 convertase component C2 (Fig. 3F) and upregulated PAI-2 (*SERPINB2*) that reduces the activity of plasmin and thereby blocks the alternative processing of C5 (Table 2) (30). Moreover, CyaA action suppressed the expression of the *NCF1* gene, the product of which plays an eminent role in NOX2-dependent superoxide generation (31, 32). Taken together, these findings suggest that CyaA toxin-exposed CD14^+^ monocytes were severely impaired in the production of chemokines that attract the inflammatory cells to infected tissue, had reduced antigen-presenting capacities and bactericidal functions, and were locked in a cell state that is insensitive to the differentiation stimulus of M-CSF.

### CyaA-treated cells contain methylated histones and abundant heterochromatin patches

**Fig 4:**
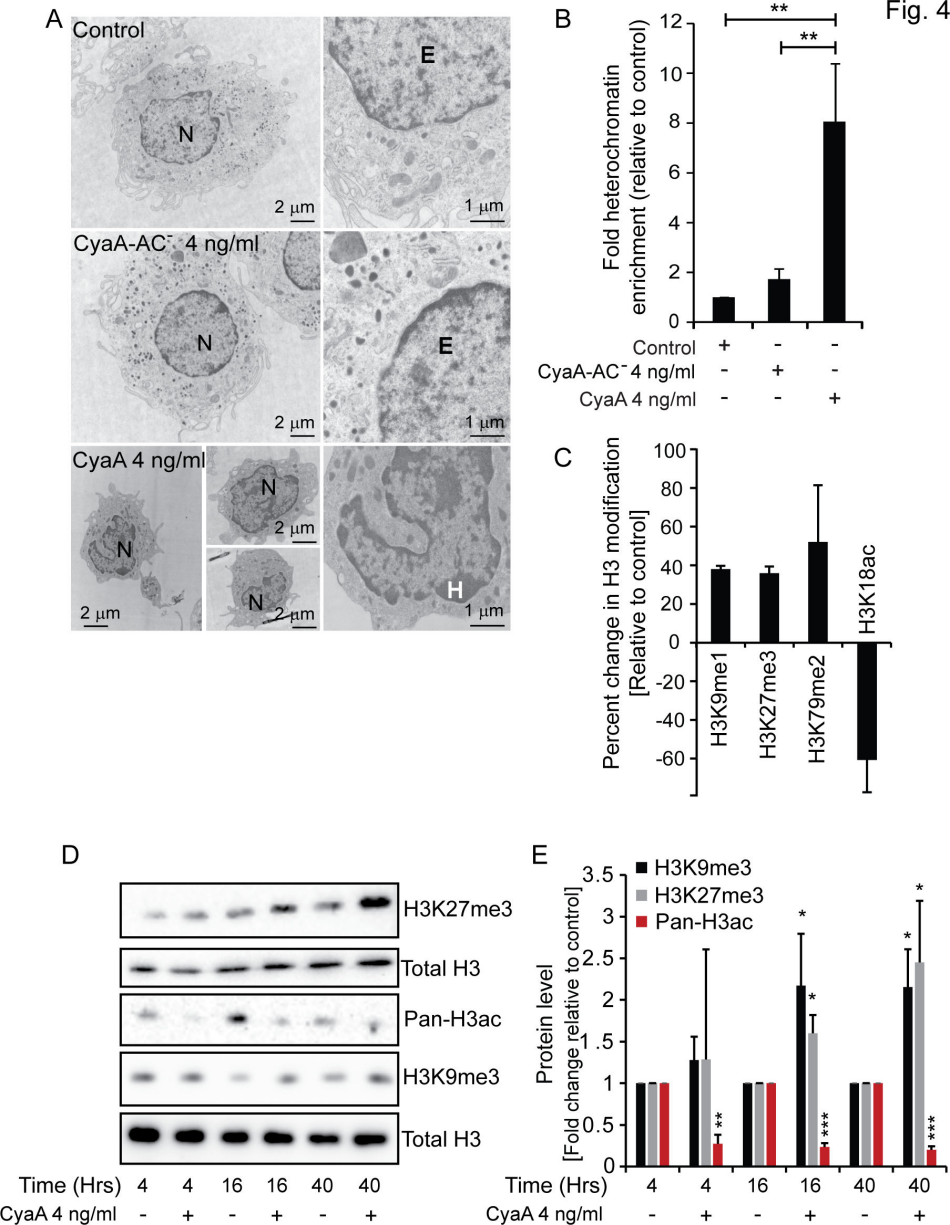
CyaA signaling drives heterochromatin formation and transcriptionally suppressive epigenetic marking of histones. TEM images of CD14^+^ monocytes cultured in DMEM containing 20 ng/mL M-CSF with or without 4 ng/mL (22 pM) CyaA or CyaA-AC^−^. Heterochromatin is visualized using uranyl acetate staining. CyaA-treated cells remain small and undifferentiated with distinct heterochromatin patches around the nuclear envelope, compared to mock-treated control or CyaA-AC^−^ toxoid-exposed cells. Representative image sections from multiple TEM images are shown. **H,** heterochromatin; **E,** euchromatin; **N,** Nucleus. (**B**) Heterochromatin-covered areas per six images per sample were quantified using ImageJ and expressed as fold-enrichment of heterochromatin in CyaA-related cells compared to mock-treated cells. **P < 0.005. (**C**) CD14^+^ monocytes were exposed to 22 pM CyaA for 40 hours in the presence of 20 ng/mL of M-CSF, and the levels of respective H3 modifications in toxin-treated cells were detected after normalization to total H3 levels using ELISA (EpiQuik Histone H3 Modification Multiplex Assay from EpigenTek). Percent change relative to the level of histone modification in control cells is shown, *n* = 2. (**D**) Detection of trimethylated residues K9me3 and K27me3 and pan-acetylation of the H3 histone. CD14^+^ monocytes were cultured with 22 pM CyaA in the presence of 20 ng/mL of M-CSF for 4, 16, and 40 hours. Histone proteins were acid-extracted and subjected to immunoblot analysis using specific antibodies. Total H3 level was used as loading control. (**E**) Quantification of the three immunoblots from panel D using ImageJ and expressed as fold change relative to signal in mock-treated control of respective samples following normalization with total H3 signal. *n* = 3, *P < 0.05, **P < 0.005, ***P < 0.0005.

The observed extent of transcriptional reprogramming indicated that CyaA action might induce alterations of the chromatin organization. Indeed, observations from transmission electron microscopy (TEM) revealed that CyaA action triggered chromatin remodeling and alteration of heterochromatin content in CD14^+^ monocytes exposed to 4 ng/mL CyaA for 5 days. Compared to cells exposed to the CyaA-AC^−^ toxoid, or to mock-treated cells cultured with 20 ng/mL M-CSF only, the CyaA-exposed cells remained undifferentiated and ~3 times smaller in size than mock or toxoid-treated cells. A striking impact of toxin action on heterochromatin content in cell nuclei was clearly apparent. Whereas nuclei of mock-treated or toxoid-exposed (4 ng/mL CyaA-AC^−^) cells contained predominantly open and decondensed euchromatin, large densely stained patches of heterochromatin were observed in locations close to the nuclear envelope in toxin-exposed cells (Fig. 4A). Quantification using ImageJ (https://imagej.net/) revealed an ~8 to 10-fold increase in heterochromatin content in patches distributed across the sections of nuclei of toxin-treated cells, as compared to control cells (Fig. 4B).

Apart from DNA methylation, the presence of histone 3 (H3) epigenetic marks, such as methylation or acetylation of specific H3 residues, is the major driver determining the condensation state of chromatin (33–40). Therefore, we investigated if CyaA action triggered transcriptionally repressive modifications of H3. Indeed, quantification of H3 modifications by residue-specific ELISA analysis of total histone extracted from cells exposed for 40 hours to 4 ng/mL CyaA (Fig. S3) revealed a substantially increased amount of mono-methylation of K9 residue of H3 (H3K9me1) that precedes further methylation by the SUV39 methyltransferase (35). Furthermore, a markedly increased dimethylation of K79 (H3K79me2), trimethylation of K27 (H3K27me3), and importantly decreased acetylation of K18 (H3K18ac) were also detected in histone extracts from toxin-treated cells (Fig. 4C). Indeed, H3K18 acetylation positively correlates with the transcriptionally permissive euchromatin state (41) and trimethylation of K27 (H3K27me3) and dimethylation of K79 of H3 (H3K79me2) are established markers of a transcriptionally repressive heterochromatin state (42–44). Furthermore, as shown in Fig. 4D and E, at 4 hours of exposure to the differentiating signal of M-CSF, a similar level of trimethylated K9 residue of H3 was still detected in toxin-exposed and control cells by immunoblot analysis of whole cell lysates. However, already at 16 hours after toxin exposure, an inhibitory effect of toxin action on M-CSF-triggered demethylation of trimethylated H3 lysine residues K9 (H3K9Me3) was apparent, compared to the differentiating mock-treated cells (Fig. 4D and E), whereas there was an increase in trimethylation of the K27 residue (H3K27me3), indicating maintenance of a transcriptionally repressive heterochromatin state. A clear toxin action-elicited difference was then observed on both H3K9me3 and on H3K27me3 at 40 hours after CyaA exposure (Fig. 4D and E). Furthermore, the pan-acetylation levels of histone H3 were assessed, and a significant decrease in pan-acetylation of H3 was observed by immunoblotting as early as 4 hours after exposure to 4 ng/mL CyaA (Fig. 4D and E). This reduction in acetylation levels remained consistent throughout the duration of the experiment, with no further significant changes detected at 16 and 40 hours (Fig. 4D and E).

These findings demonstrate that exposure to already very low amounts of CyaA toxin (22 pM) triggers significant epigenetic modifications in monocytes, revealing the mechanism by which CyaA toxin action inhibits the M-CSF-elicited transcriptional program required for monocyte differentiation to macrophages.

### HDAC inhibitor trichostatin-A and G9a methyltransferase inhibitor UNC 0631 alleviate CyaA-elicited effects on gene expression and the block of monocyte to macrophage transition

**Fig 5:**
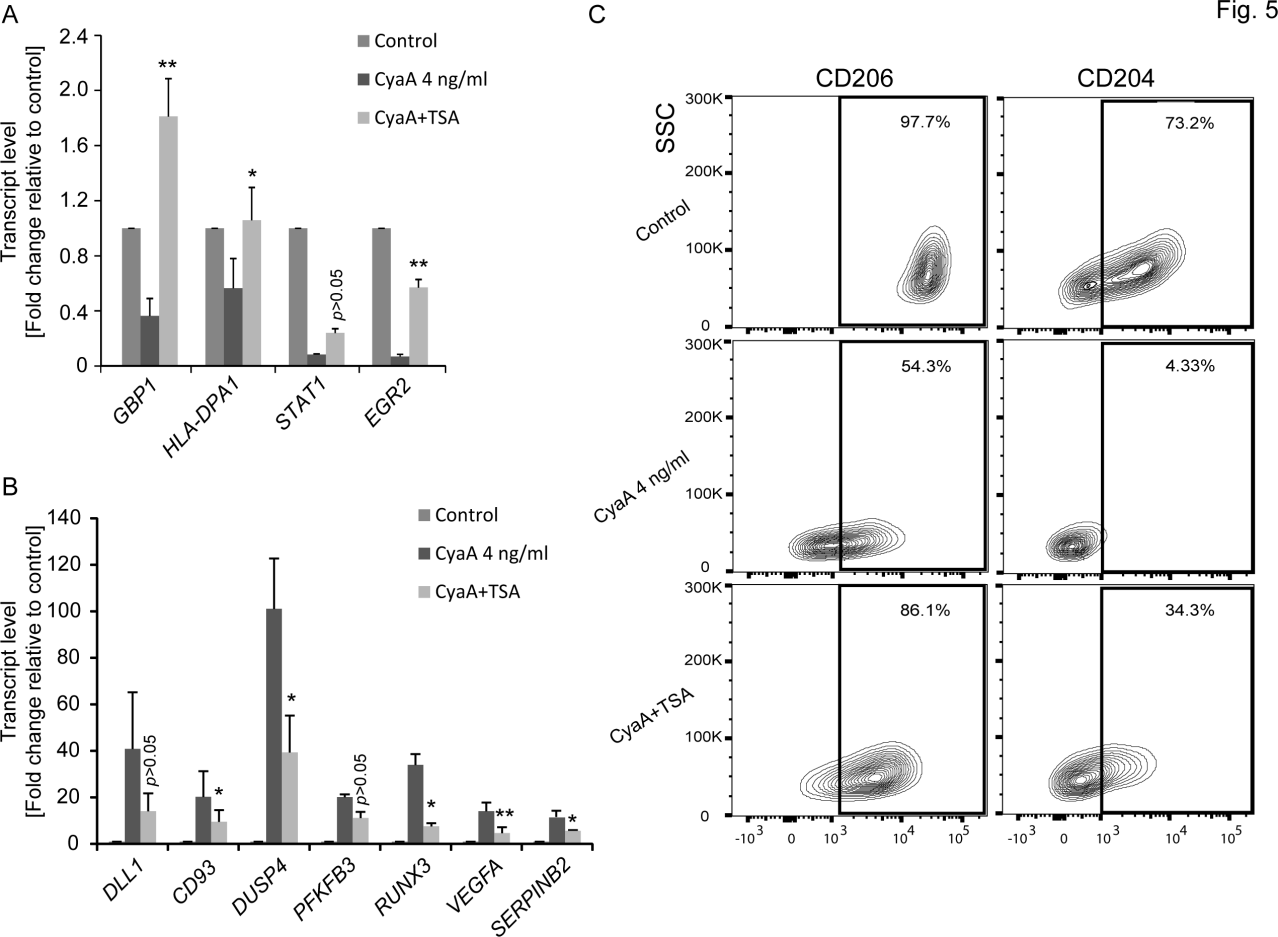
TSA alleviates the CyaA-elicited transcriptional reprogramming and arrest of monocyte differentiation. (**A, B**) Transcript levels of indicated genes of CD14^+^ monocytes treated with 4 ng/mL of CyaA in the presence or absence of the HDAC inhibitor TSA (160 nM) were quantified using qPCR. (**A**) Downregulated genes and (**B**) upregulated genes. Gene expression was normalized to β-actin and β-2 microglobulin mRNA levels. Results are presented as fold change relative to control (*n* = 4, *P < 0.05, **P < 0.005). (**C**) CD14^+^ monocytes cultured for 5 days with the differentiation stimulus of human M-CSF (20 ng/mL) along with CyaA (4 ng/mL) in the presence or absence of 160 nM TSA were analyzed for the levels of macrophage surface markers CD206 and CD204 by FACS. Contour plots representative of three independent experiments are shown.

Deacetylation is a prerequisite for the transcription-silencing methylation of H3. Therefore, we used the class II histone deacetylase (HDAC II) inhibitor trichostatin-A (TSA) to corroborate the mechanism by which CyaA-elicited signaling triggered chromatin remodeling. qPCR analysis revealed that inhibition of HDAC II in CD14^+^ monocytes exposed to CyaA (4 ng/mL) for 40 h in the presence of 160 nM TSA alleviated the transcriptional downregulation of selected immunity-related genes (Fig. 5A), such as *GBP1, HLA-DPA1, STAT1,* and *EGR2*. At the same time, TSA inhibited the CyaA-triggered upregulation of transcription *DUSP4* involved in the deactivation of the MAPK pathways, limiting cytokine production and interfering with monocyte differentiation into macrophages (*SERPINB2, DLL1*), or involved in intercellular adhesion (*CD93*), inflammatory monocyte proliferation (*PFKB3*), and regulation of cell death and angiogenesis (*RUNX3, VEGFA*), respectively (Fig. 5B). In line with these observations, HDAC inhibition by TSA alleviated, in part, the CyaA-induced block of M-CSF-driven monocyte to macrophage differentiation, as documented by the increase in the numbers of cells expressing the macrophage markers CD206 and CD204 (Fig. 5C).

**Fig 6:**
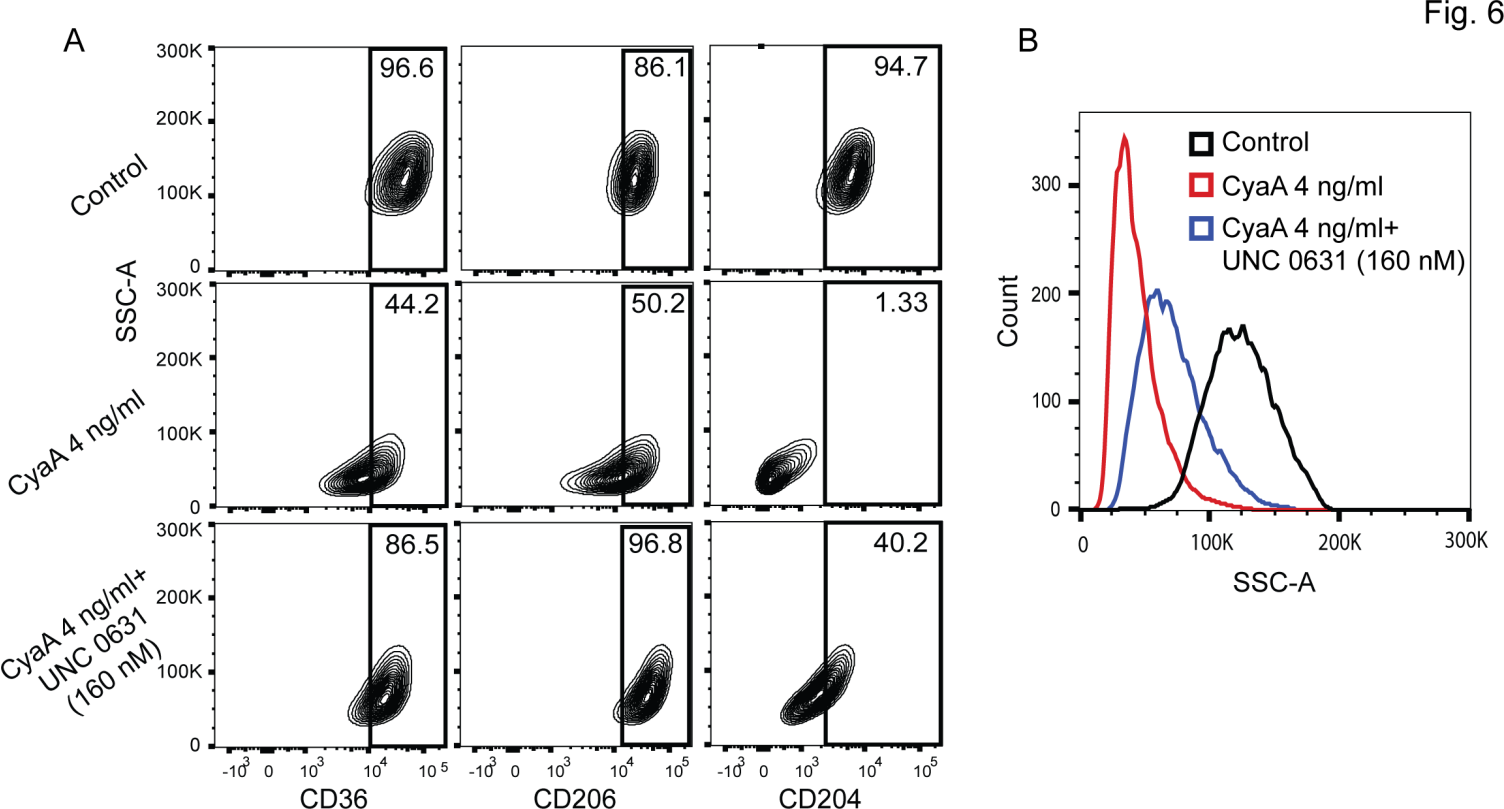
Inhibition of the G9a methyltransferase relieves the CyaA-elicited monocyte differentiation block. (**A**) CD14^+^ monocytes were exposed to 22 pM CyaA for 8 days in the presence or absence of the G9a methyltransferase inhibitor UNC 0631 (160 nM). Monocyte differentiation to macrophages was assessed by analyzing the expression of macrophage markers CD36, CD206, and CD204. The representative contour plot illustrates the differentiation status of the monocytes under these conditions (*n* = 3). (**B**) Side scatter histograms of control and CyaA-treated cells, with or without UNC 0631 (160 nM), are shown; the histograms depict the changes in intracellular complexity (*n* = 3).

The increased methylation at several H3 residues (c.f. Fig. 4) is a prerequisite for the formation and spread of condensed chromatin in cell nuclei (40). We, thus, examined if inhibition of methyltransferase activity by a set of known inhibitors would alleviate the block of monocyte differentiation imposed by CyaA toxin action. Of the several tested compounds (data not shown), the UNC 0631 inhibitor specific for the G9a methyltransferase, known to methylate both H3K9 and H3K27 (45–47), efficiently relieved the CyaA-elicited block of monocyte differentiation (Fig. 6A and B). The addition of 160 nM UNC 0631 alleviated the block of monocyte differentiation elicited by 22 pM CyaA and yielded a significant increase in the number of M-CSF-differentiated cells expressing the CD36, CD206, and CD204-macrophage markers (Fig. 6A). Moreover, compared to monocytes treated with CyaA alone, the CyaA-exposed cell cultures with the 160 nM G9a inhibitor UNC 0631 exhibited notably higher flow cytometry side scatter values, reflecting increased intracellular complexity, which is a macrophage feature (Fig. 6B). However, the inhibition of G9a activity under the used condition was likely not complete, as the monocytes took 8 days to differentiate into macrophages in the presence of 160 nM UNC 0631 and 22 pM CyaA. These data, nevertheless, clearly indicate that inhibition of G9a-mediated methylation of K9 and K27 residues of H3 was crucial for overcoming the CyaA-imposed downregulation of the gene products that play a key role in monocyte differentiation into macrophage cells.

**Fig 7:**
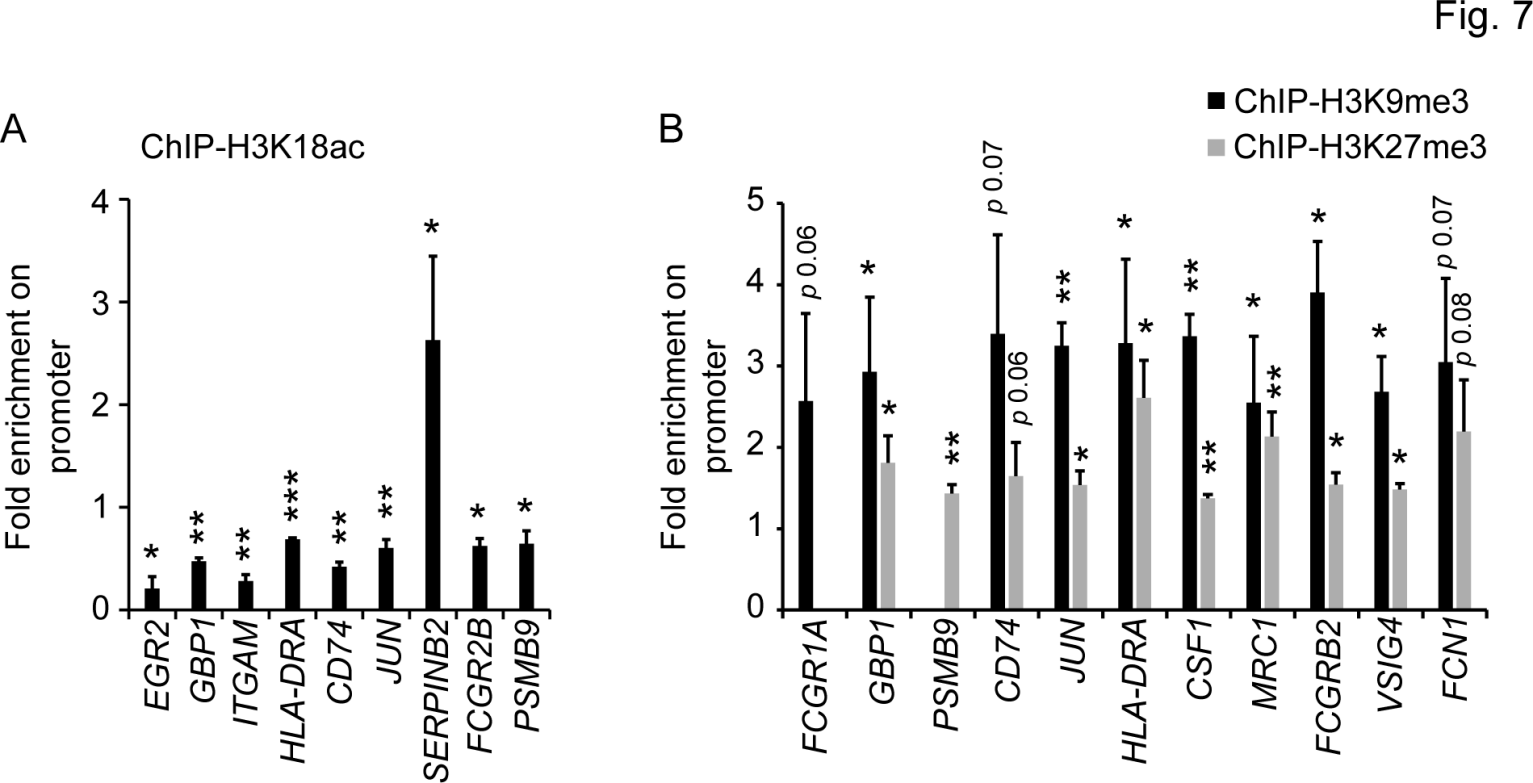
H3 methylation and acetylation at specific promoters. ChIP analysis of H3 acetylation (**A**) and methylation (**B**) at promoters of selected genes involved in immune response and monocyte differentiation. CD14^+^ monocytes were treated with 22 pM CyaA for 40 hours and ChIP assay was performed using an antibody against H3K18ac (**A**) or against H3K9me3 and H3K27me3 (**B**) to enrich for histone H3 acetylated at lysine 18 (**A**), or for H3 trimethylated at lysine 9 and lysine 27 residues (**B**), respectively. Promoter regions of the enriched DNA fragments were quantified by qPCR, and the results are presented as fold change following normalization to sheared chromatin of the input sample, *n* = 3, *P < 0.05, **P < 0.005.

To further elucidate the impact of H3 acetylation/methylation balance on CyaA-mediated deregulation of gene expression, chromatin immunoprecipitation (ChIP) analysis was performed on CD14^+^ monocytes exposed to 22 pM CyaA for 40 hours. The DNA fragments enriched by immunoprecipitation of chromatin complexes with antibodies specific for the modified H3K18ac (Fig. 7A) and H3K9me3 or H3K27me3 residues (Fig. 7B) were quantified by qPCR using a set of selected promoter-specific primers (Table S3). As documented in Fig. 6C decreased pull-down yield due to decreased H3K18 acetylation at several specific gene promoters of downregulated genes was observed, whereas an increased acetylation of H3K18 was found at the promoter of the highly transcribed *SERPINB2* gene (Fig. 7A). Concurrently, an increase in methylation at H3K9 and H3K27 residues was detected at numerous promoters of genes downregulated upon cell exposure to CyaA (Fig. 7B). Notably, reduced H3K18 acetylation at promoters of genes encoding the transcription factors *JUN*, *EGR2* and simultaneous increase in trimethylated H3K9me3 or H3K27me3 residues at promoters of the *JUN*, *CSF1* genes was observed. Altogether, these data underscore the crucial role of H3 acetylation and methylation in the epigenetic mechanism of CyaA-elicited downregulation of the pro-differentiation gene expression program in monocytes exposed to CyaA toxin.

## DISCUSSION

**Fig 8:**
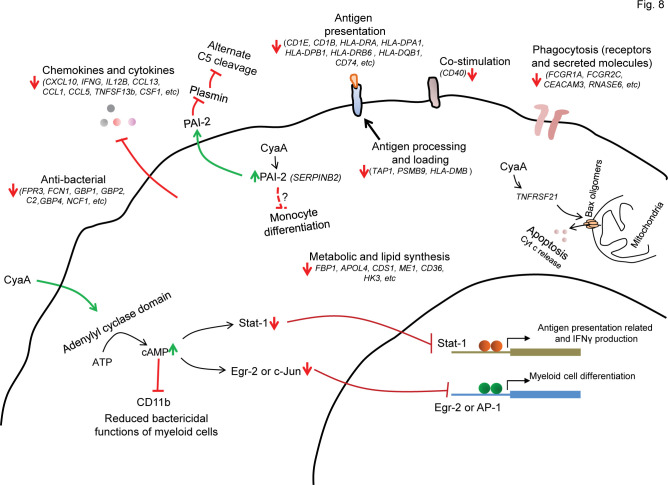
Signaling of very low amounts of *B. pertussis* CyaA toxin causes transcriptional reprogramming of CD14^+^ monocytes. Numerous cellular processes are altered upon exposure of CD14^+^ monocytes to as little as 4 ng/mL (22 pM) CyaA for 40 hours. CyaA-elicited cAMP signaling reduces chemokine and cytokine gene expression and the expression of genes involved in antigen processing and presentation to T cells, such as *PSMB9, TAP1, HLA-DMB, CD1E, HLA-DRA, CD74*. CyaA-exposed cells are metabolically affected, as deduced from decreased expression of the *APOL4, ME1, CD36,* and *HK3* genes involved in the provision of essential lipids during monocyte differentiation into macrophages (55). Complement-based bactericidal functions are compromised due to low *C2* level, whereas higher expression of PAI-2 blocks the alternate way of plasmin-mediated C5 cleavage (30). Phagocytosis is primarily affected by the reduction of expression levels of the phagocytic receptor *FCGR1A* and the phagocytic cup component *ASAP2* (28). Lower levels of proteins encoded by the *IFNG*, *ITGAM,* and *NCF1* genes further compromise the bactericidal functions of CyaA-exposed cells. Toxin action decreases the expression of positive regulators of monocyte differentiation, such as EGR2, and the AP-1 transcription factor components. CyaA triggers Bax association with mitochondria, likely due to the increased levels of *TNFRSF21* transcription and Bim stabilization, as these molecules can trigger Bax oligomerization at the outer mitochondrial membrane and elicit release of the pro-apoptotic mitochondrial intermembrane molecules, such as the cytochrome *c* (Cyt *c*) (13, 56).

We report that CyaA toxin action blocks monocyte to macrophage transition through epigenetic reprogramming of monocytes. When monocytes differentiate into macrophages, their cycle is arrested, and instead of proliferation, the cell volume grows to attain the large macrophage cell type. This transition of primary circulating monocytes into macrophages is accompanied by remarkable changes in morphology and cell function, which requires the products of a number of newly transcribed gene sets (48, 49). We show by RNAseq transcriptional profiling that the cAMP-mediated signaling of very low amounts of *B. pertussis* CyaA toxin provokes the downregulation of transcription of numerous genes, as schematically summarized in Fig. 8. We report that CyaA elicits epigenetic modifications that are known to block the opening of chromatin for transcription and promote the formation of condensed heterochromatin that can restrict the access of the transcriptional machinery to specific promoter and enhancer sequences. This CyaA action appears to be mediated by the activation of histone deacetylation, followed by methylation of the lysine residues of histone H3 (c.f. Fig. 4), required for bridging of adjacent nucleosomes and heterochromatin formation (35, 50). Indeed, CyaA action on monocytes resulted in deacetylation of H3K18, known to cause a block of gene expression (41). Moreover, the observed CyaA-triggered trimethylation of H3K27 and dimethylation of H3K79 would instigate the formation of closed chromatin and prevent the association of transcription factors with the respective promoter regions (42, 51). Furthermore, the observed increase in the amounts of monomethylated H3K9 (H3K9me1, c.f. Fig. 4C), recognized by the SUV39 methyltransferase (52, 53), yielded trimethylation of H3K9, with the accumulation of H3K9me3 being a hallmark of heterochromatin formation and gene silencing (54).

The CyaA/cAMP signaling-elicited block of monocyte differentiation appears to be downstream of G9a methyltransferase (c.f. Fig. 6), as the G9a methyltransferase inhibitor UNC 0631 alleviated the CyaA effect on differentiation block, likely due to upregulation of G9a expression by the cAMP-activated protein kinase A (PKA) signaling axis (57). Furthermore, previous findings suggested a role for PKA in blocking the nuclear export of histone deacetylases HDAC4 and HDAC5 (58, 59). This is in line with our earlier observation that CyaA toxin action promoted the dephosphorylation of HDAC5 and may block its nuclear export (60). Furthermore, zinc ions are essential for HDAC activity (61, 62) and CyaA action yielded upregulation of the zinc ion importer gene *SLC39A14* (c.f. Table 2 and Fig. 2B). Conversely, the presence of the HDAC inhibitor TSA, which blocks HDAC activity by chelating zinc ions (63), alleviated, in part, the toxin-mediated suppression of transcription of some genes and the block of CyaA-mediated M-CSF-driven monocyte differentiation (Fig. 5). On the contrary, CyaA action also upregulated transcription of certain groups of genes, and this was reversed, in part, by TSA treatment (c.f. Fig. 5B). A plausible explanation of this seemingly contradictory observation would be that the activity of some transcriptional repressors is maintained by acetylation (64). Hence, their HDAC-mediated deacetylation would convert them to transcriptional activators (65). It is plausible to speculate that through cAMP signaling, the CyaA activity provokes retention of some of the HDACs in the nucleus and thereby enhances the deacetylation of histones and some non-histone proteins. This would facilitate the assembly of heterochromatin patches across the genome while activating, at the same time, the selective expression of genes that negatively regulate cell differentiation, such as *SERPINB2* (PAI-2) (25).

Another layer of CyaA-triggered transcriptional regulation and inhibition of monocyte differentiation would depend on the modulation of the activity of transcriptional factors AP-1 and EGR2. We have previously shown that CyaA/cAMP-mediated upregulation of tyrosine phosphatase SHP-1 activity results in dephosphorylation of the c-fos component of the AP-1 transcription factor, which blocks its activity (66). Moreover, cAMP signaling was shown to attenuate the expression of EGR2, as well as of the JUN dimerizing partners, which are involved in the formation of the active AP-1 transcription factor (67, 68). Since both EGR2 and AP-1 are known to be involved in the transcriptional program required for monocyte transition to macrophage cells (23, 69), their poor expression due to the reduced K18 acetylation and enhanced trimethylation at respective gene promoters in CyaA-treated cells would further contribute to the block of monocyte differentiation (c.f. Fig. 7A and B). Furthermore, CyaA/cAMP-promoted upregulation of the negative regulators of monocyte differentiation, encoded by the genes *CSNK1E* and *SERPINB2* (PAI-2) (c.f. Fig. 2 and 3), would yield locking of the CD14^+^ monocyte in an undifferentiated state (15, 25, 26, 70, 71).

Differentiation of incoming circulating monocytes into bactericidal macrophages represents a key element of innate mucosal immune protection from bacterial infection. Previous work revealed that the action of *B. pertussis* CyaA at low concentrations blocks the capacity of macrophage cells to phagocytose complement-opsonized particles (12, 15), which is an important mechanism of immune protection of the mucosa in naïve infants lacking specific antibodies to *B. pertussis* antigens. The transcriptional profiling work reported here reveals that CyaA toxin action can further attenuate the macrophage-mediated immune defense of host mucosa by blocking the transition of chemoattracted monocytes into bactericidal macrophage cells. Hence, exposure to CyaA blocks the employment of a number of immunity-related functions of macrophages, yielding the here reported downregulation of expression of phagocytic receptor genes *FCGR1A* and the phagocytic cup component gene *ASAP2* or the upregulation of *DUSP4* which inactivates the MAP kinase signaling (c.f. Fig. 3F). Our results suggest new ways of blocking *B. pertussis*-mediated reprogramming of monocytes by “epigenetic” drugs that are currently undergoing clinical trials for cancer therapy. It will be of interest to examine their effects on the outcome of *B. pertussis* infection in suitable animal models of catarrhal pertussis (72).

## MATERIALS AND METHODS

### Antibodies and reagents

EpiQuik histone H3 modification multiplex assay kit (Cat. No. P-3100) and EpiQuik total histone extraction kit (Cat. No. OP-0006) were from Epigentek. Anti-CD206 (clone MR6F3) and anti-CD204 (clone PSL204) were purchased from Invitrogen; anti-CD14 (MEM-18), anti-CD11b (clone ICRF44), anti-CD36 (clone TR9), and anti-fcgr1a (clone 10.1) were obtained from Exbio. anti-Fcgr2B (clone 19072) from Bio-Techne; polyclonal anti-H3K27me3 and anti-H3K9me3 were from Active Motif. Recombinant Human M-CSF (Cat. No. #300-25) was from PeproTech, and CD14 MicroBeads (Cat. No. #130-050-201) were from Miltenyi Biotec. DMEM was from Sigma-Aldrich (Cat. No. #D6429), Trichostatin-A and UNC 0631 were from MedChemExpress and Ficoll from GE Health Care.

### CyaA and CyaA-AC^−^ purification

Recombinant CyaA and CyaA-AC^−^ proteins were produced in *E. coli* XL1-Blue (Stratagene, La Jolla, CA) expressing *cyaC* and *cyaA* genes from the pT7CACT1 plasmid (17) and the proteins purified by a combination of ion-exchange chromatography and hydrophobic chromatography as described previously (17). Endotoxin was removed from protein preparations by on-column washing of the resin-bound proteins with 60% isopropanol (73), and the endotoxin level was analyzed using chromogenic limulus amebocyte lysate assay kit (QCL-1000; Lonza, Walkersville, MD). The level of endotoxin was less than 0.1 endotoxin units (EU) of *E. coli* LPS per 1 µg of protein.

### Total RNA extraction and library construction for deep sequencing

CD14^+^ monocytes were seeded in 6-well plates at a density of 1  ×  10^6^ cells/mL in 5 mL of DMEM with 10% FCS and 20 ng/mL of human recombinant M-CSF (PeproTech). After an hour of cell resting, CyaA toxin or the CyaA-AC^−^ toxoid was added to a final concentration of 4 ng/mL (22 pM), and the culture was continued for 40  hours without change of media at 37°C in a humidified 5% CO_2_ atmosphere. Culture supernatant was aspirated, cells were washed once with ice-cold PBS, and total RNA was extracted using the RNA-blue reagent (Top-Bio, Czech Republic) according to manufacturer’s instructions. The amount of RNA was quantified by microspectrophotometry (DeNovix), and RNA quality was assessed using an Agilent Bioanalyzer. High-quality RNA with a RIN score >7 was used for cDNA library preparation from total mRNA using NEB Next Ultra II Directional RNA with polyA mRNA isolation module kit. Tagged NGS libraries were loaded onto NovaSeq 6000 (Illumina) with a sequence output of 20 M reads per sample.

### Quantitative PCR

cDNA was prepared from total RNA samples of CD14^+^ monocytes using high-capacity cDNA reverse transcription kit (Applied Biosystems) and used for qPCR amplification using gene-specific primers (Table S2). The CT values were normalized to levels of β-actin and β-2 microglobulin gene transcripts, and the relative expression levels of genes were compared to the expression levels of respective genes in mock-treated control cells.

### CD14^+^ monocyte separation from PBMCs

Human peripheral blood mononuclear cells (PBMCs) were purified from leukopaks obtained after processing of blood of anonymous healthy donors at the Blood Transfusion Center of the Thomayer Hospital in Prague, Czech Republic. Cells were concentrated using density gradient centrifugation over Ficoll-Paque (GE Healthcare) according to the Miltenyi Biotec protocol. PBMCs were washed with ice-cold PBS containing 1 mM EDTA, the cell pellet was resuspended into 0.5 mL of PBS with 0.5% BSA, and 1 mM EDTA (bead buffer) and 40 µL of anti-human CD14 microbeads were added per sample prior to incubation at 4°C for 40 min. The cells were pelleted and washed with bead buffer, and CD14^+^ monocytes were separated using Possel_s program of the MACS cell separation instrument (Miltenyi Biotech). The CD14^+^ cells were counted, and purity was assessed by fluorescence-activated cell sorting (FACS) using a fluorophore labeled CD14 antibody. The obtained cells were highly homogeneous, exhibiting more than 95% purity (Fig. S1).

### Analysis of monocyte differentiation and toxin treatment

Purified CD14^+^ monocytes were cultured in DMEM containing 10% FCS and 20 ng/mL of M-CSF (differentiation media), incubated with or without CyaA toxin or the CyaA-AC^−^ toxoid (4 ng/mL) at 37°C in a humidified 5% CO_2_ atmosphere for the indicated time. Where appropriate, cells were pre-incubated with trichostatin-A (160 nM) or UNC 0631 (160 nM) prior to toxin addition. The culture media was replaced every 48 hours with fresh differentiation media along with toxin or toxoid. Wherever applicable, the TSA or UNC 0631 inhibitors were added at the indicated concentrations, and the cells were incubated for the indicated time. Cells were next washed with ice-cold Hanks’ balanced salt solution (HBSS) and detached with Accutase solution (Cat. No. A6964), washed with HBSS, pelleted, and resuspended in HBSS with 1% FCS. The viability of cells at the end of the incubation experiments was systematically analyzed by staining with Hoechst 33258 (1 μg/mL). The cells were next incubated with fluorophore-labeled antibodies against indicated surface markers and analyzed using the LSR II flow cytometer (BD Biosciences). Cytometric data were analyzed using FlowJo (Tree Star, Ashland, OR). For immunoblotting, cell lysates were acid-extracted using the EpiQuik total histone extraction kit from EpigenTek according to the manufacturer’s instructions. Briefly, the cells were treated with 4 ng/mL of CyaA and incubated at 37°C for various time points. Following incubation, cells were immediately fixed with 0.8% formaldehyde for 5 minutes at room temperature, and the remaining reactive aldehyde groups were quenched by the addition of 125 mM glycine. The cells were washed three times with ice-cold PBS and permeabilized with 0.2% Triton X-100 for 15 minutes on ice. The cell membrane was disrupted by three cycles of high-speed vortexing for 20 seconds, followed by centrifugation at 5,000 RPM for 5 minutes. The pellet was washed with ice-cold PBS and dissolved in the acidic lysis buffer from the EpiQuik Total Histone Extraction Kit (EpigenTek). Following incubation for 30 minutes on ice, the samples were heated at 65°C for 2 hours to reverse protein crosslinking. The extracted proteins were separated on a 12.5% SDS-PAGE gel and transferred onto a nitrocellulose membrane. The respective proteins were then detected using specific primary and HRP-conjugated secondary antibodies and visualized using enhanced chemiluminescent substrate (SuperSignal West Femto, ThermoFisher) in a GBOX-Chemi-XRQ-E system (Syngene, Frederick, MD, USA). Wherever applicable, the luminescence signal was quantified using ImageJ software (https://imagej.nih.gov/ij/).

### Chromatin immunoprecipitation

CD14^+^ monocytes were cultured in DMEM supplemented with 10% fetal calf serum (FCS) and 20 ng/mL of M-CSF. Cells were incubated at 37°C in a CO_2_ incubator for 40 hours, with or without the addition of the CyaA toxin or its CyaA-AC^−^ toxoid (22 pM, 4 ng/mL). ChIP was next performed using antibodies against H3K9me3, H3K27me3, and H3K18Ac by utilizing a kit from Active Motif (ChIP-IT Express Enzymatic Kit, Cat. No. 53009) according to manufacturer’s instructions. Briefly, cells were fixed immediately after removal from the 37°C CO₂ incubator by treatment with 0.8% formaldehyde for 7 minutes at room temperature, and the remaining free aldehyde groups were quenched with 125 mM glycine. Cells were next washed three times with ice-cold PBS, and the cell pellet was resuspended in 600 µL of lysis buffer from the kit supplemented with a cOmplete, Mini, EDTA-free Protease Inhibitor Cocktail (Roche, Cat.alog number: 11836170001), and 1 mM PMSF. The suspension was incubated on ice for 15 minutes to allow cell lysis. Subsequently, the cells were transferred to an ice-cold Dounce homogenizer and lysed by performing 60 strokes. Cell nuclei were collected by centrifugation in Eppendorf tubes at 5,000 RPM for 10 minutes at 4°C. The supernatant was carefully discarded, and the nuclear pellet was resuspended in 200 µL of digestion buffer containing micrococcal nuclease (final concentration: 1666 U/mL), cOmplete, Mini (Roche, Catalog. No. 11836170001) protease inhibitor cocktail, and 1 mM PMSF. The suspension was incubated at 37°C for 10 minutes to allow chromatin digestion. To stop the digestion reaction, 5 µL of ice-cold 0.5 M EDTA was added, and the samples were chilled on ice for 10 minutes. The samples were centrifuged at 15,000 RPM for 10 minutes in a chilled microcentrifuge, and the chromatin-containing supernatant was collected for subsequent use. Ten microliters of aliquots of sheared chromatin was saved as the input sample. For chromatin de-crosslinking, 50 µL of sheared chromatin was treated with 150 µL of distilled water, followed by the addition of 10 µL of 5 M NaCl, and samples were heated at 65°C for 4 hours to reverse crosslinking. 1 µL of RNase A was then added, and the samples were incubated for 15 minutes at 37°C. 10 µL of proteinase K was added next, and the samples were incubated for an additional 1.5 hours at 42°C. The chromatin was purified using the Monarch PCR and DNA Cleanup Kit (New England Biolabs, Catalog. No. T1030L) and subsequently analyzed by gel electrophoresis on a 2% agarose gel. Chromatin was then subjected to ChIP, and the enriched fragments were collected following the manufacturer’s protocol. The DNA fragments obtained from the ChIP assays were analyzed for enriched promoter fragments using specific primer sets (Table S3), and the enrichment was quantified by qPCR upon normalization to sample input.

### Transmission electron microscopy

CD14^+^ monocytes differentiated in the presence or absence of CyaA or CyaA-AC^−^ (4 ng/mL) for 5 days were washed three times with ice-cold PBS and fixed with 2% glutaraldehyde–PBS on ice for 2 hours. The fixed cells were washed three times with ice-cold PBS prior to post-fixing with 0.5% osmium tetroxide-PBS overnight at 4°C. Post-fixed cells were dehydrated by using ethanol series and embedded in epoxy resin (EMBed-812 Embedding kit; Electron Microscopy Sciences). Ultrathin sections were contrasted using uranyl acetate and lead citrate (Reynolds 1963). Contrasted sections were examined in an FEI Morgagni 268(D) electron microscope (FEI, Brno, Czech Republic) at 80 kV. Digital images were recorded with MegaViewIII slow scan camera and processed by AnalySis 3.2 (Olympus Soft Imaging Solutions GmbH, Münster, Germany) using standard software modules (shading correction, digital contrast enhancement).

### RNAseq data analysis

Sequencing reads were processed with Nextflow version 22.10.4 (74) using the nf-core/RNAseq pipeline version 3.10.1 (https://zenodo.org/records/4323183) with default settings. Differential expression was assessed against the null hypothesis of absolute log fold change <1 using the DESeq2 R package version 1.38.3 (75), using the apeglm package version 1.20.0 for shrinkage (76). Gene set enrichment was computed with clusterProfiler version 4.6.0 (20) and fgsea version 1.24.0 (77) packages. Code and data to reproduce the analysis are available at Zenodo: https://zenodo.org/records/8189564.

### Determination of histone modification by ELISA

CD14^+^ monocytes were cultured in DMEM supplemented with 20 ng/mL human recombinant M-CSF (Peprotech), and cells were treated with CyaA (4 ng/mL) for the indicated time. Total histone proteins were extracted using the EpiQuik total histone extraction kit (EpigenTek) as above. Multiple histone H3 modifications were analyzed by ELISA (EpiQuik histone H3 modification multiplex assay kit) according to the manufacturer’s instructions.

### Quantitative detection of IL-10 by ELISA

Purified CD14^+^ monocytes were cultured in DMEM supplemented with 10% FCS. Cells were seeded at a density of 1 × 10^6^ cells/mL in 12-well plate. Monocytes were treated with 4 ng/mL of CyaA for 40 hours at 37°C in a humidified atmosphere containing 5% CO₂. The concentration of IL-10 in the supernatants was measured using an IL-10 ClinMax Human IL-10 ELISA Kit (Cat. No. CRS-B005) according to the manufacturer’s instructions. The concentration of IL-10 in the samples was determined by comparing the absorbance values to a standard curve generated using known concentrations of IL-10. Results were expressed as pg/mL of IL-10.

### Statistics

Statistical analysis was performed using the GraphPad embedded paired *t* test algorithm.

## SUPPLEMENTAL MATERIAL

Fig. S1 mbio.00138-25-s0001.tif FACS analysis of purified CD14+ monocytes.

Fig. S2 mbio.00138-25-s0002.tif Comparative qPCR analysis after cell exposure to CyaA or the CyaA-AC- toxoid.

Fig. S3 mbio.00138-25-s0003.tif Analysis of various histone H3 residue modifications.

Table S1 mbio.00138-25-s0004.docx List of all DEG.

Table S2 mbio.00138-25-s0005.docx Primers used in this study.

Table S3 mbio.00138-25-s0006.docx Primers used for ChIP-qPCR.

ASM does not own the copyrights to Supplemental Material that may be linked to, or accessed through, an article. The authors have granted ASM a non-exclusive, world-wide license to publish the Supplemental Material files. Please contact the corresponding author directly for reuse.

## References

[1] Mattoo S, Cherry JD. 2005. Molecular pathogenesis, epidemiology, and clinical manifestations of respiratory infections due to Bordetella pertussis and other Bordetella subspecies. Clin Microbiol Rev 18:326–382. doi:10.1128/CMR.18.2.326-382.2005 15831828 PMC1082800

[2] Belcher T, Dubois V, Rivera-Millot A, Locht C, Jacob-Dubuisson F. 2021. Pathogenicity and virulence of Bordetella pertussis and its adaptation to its strictly human host. Virulence 12:2608–2632. doi:10.1080/21505594.2021.1980987 34590541 PMC8489951

[3] Melvin JA, Scheller EV, Miller JF, Cotter PA. 2014. Bordetella pertussis pathogenesis: current and future challenges. Nat Rev Microbiol 12:274–288. doi:10.1038/nrmicro3235 24608338 PMC4205565

[4] Carbonetti NH. 2016. Pertussis leukocytosis: mechanisms, clinical relevance and treatment. Pathog Dis 74:ftw087. doi:10.1093/femspd/ftw087 27609461 PMC5761200

[5] Carbonetti NH. 2015. Contribution of pertussis toxin to the pathogenesis of pertussis disease. Pathog Dis 73:ftv073. doi:10.1093/femspd/ftv073 26394801 PMC4626579

[6] Ahmad JN, Sebo P. 2021. Bacterial RTX toxins and host immunity. Curr Opin Infect Dis 34:187–196. doi:10.1097/QCO.0000000000000726 33899753

[7] Linhartová I, Bumba L, Mašín J, Basler M, Osička R, Kamanová J, Procházková K, Adkins I, Hejnová-Holubová J, Sadílková L, Morová J, Sebo P. 2010. RTX proteins: a highly diverse family secreted by a common mechanism. FEMS Microbiol Rev 34:1076–1112. doi:10.1111/j.1574-6976.2010.00231.x 20528947 PMC3034196

[8] Fedele G, Schiavoni I, Adkins I, Klimova N, Sebo P. 2017. Invasion of dendritic cells, macrophages and neutrophils by the Bordetella adenylate cyclase toxin: a subversive move to fool host immunity. Toxins (Basel) 9:293. doi:10.3390/toxins9100293 28934122 PMC5666340

[9] Novak J, Cerny O, Osickova A, Linhartova I, Masin J, Bumba L, Sebo P, Osicka R. 2017. Structure–function relationships underlying the capacity of Bordetella adenylate cyclase toxin to disarm host phagocytes. Toxins (Basel) 9:300. doi:10.3390/toxins9100300 28946636 PMC5666347

[10] Cerny O, Anderson KE, Stephens LR, Hawkins PT, Sebo P. 2017. cAMP signaling of adenylate cyclase toxin blocks the oxidative burst of neutrophils through epac-mediated inhibition of phospholipase C activity. J Immunol 198:1285–1296. doi:10.4049/jimmunol.1601309 28039302

[11] Eby JC, Gray MC, Hewlett EL. 2014. Cyclic AMP-mediated suppression of neutrophil extracellular trap formation and apoptosis by the Bordetella pertussis adenylate cyclase toxin. Infect Immun 82:5256–5269. doi:10.1128/IAI.02487-14 25287922 PMC4249293

[12] Kamanova J, Kofronova O, Masin J, Genth H, Vojtova J, Linhartova I, Benada O, Just I, Sebo P. 2008. Adenylate cyclase toxin subverts phagocyte function by RhoA inhibition and unproductive ruffling. J Immunol 181:5587–5597. doi:10.4049/jimmunol.181.8.5587 18832717

[13] Ahmad JN, Cerny O, Linhartova I, Masin J, Osicka R, Sebo P. 2016. cAMP signalling of Bordetella adenylate cyclase toxin through the SHP-1 phosphatase activates the BimEL-Bax pro-apoptotic cascade in phagocytes. Cell Microbiol 18:384–398. doi:10.1111/cmi.12519 26334669

[14] Gordon S, Taylor PR. 2005. Monocyte and macrophage heterogeneity. Nat Rev Immunol 5:953–964. doi:10.1038/nri1733 16322748

[15] Ahmad JN, Holubova J, Benada O, Kofronova O, Stehlik L, Vasakova M, Sebo P. 2019. Bordetella adenylate cyclase toxin inhibits monocyte-to-macrophage transition and dedifferentiates human alveolar macrophages into monocyte-like cells. mBio 10:e01743-19. doi:10.1128/mBio.01743-19 31551332 PMC6759761

[16] Ahmad JN, Sebo P. 2020. Adenylate cyclase toxin tinkering with monocyte-macrophage differentiation. Front Immunol 11:2181. doi:10.3389/fimmu.2020.02181 33013916 PMC7516048

[17] Osicka R, Osicková A, Basar T, Guermonprez P, Rojas M, Leclerc C, Sebo P. 2000. Delivery of CD8(+) T-cell epitopes into major histocompatibility complex class I antigen presentation pathway by Bordetella pertussis adenylate cyclase: delineation of cell invasive structures and permissive insertion sites. Infect Immun 68:247–256. doi:10.1128/IAI.68.1.247-256.2000 10603395 PMC97128

[18] Sander J, Schmidt SV, Cirovic B, McGovern N, Papantonopoulou O, Hardt A-L, Aschenbrenner AC, Kreer C, Quast T, Xu AM, et al.. 2017. Cellular differentiation of human monocytes is regulated by time-dependent interleukin-4 signaling and the transcriptional regulator NCOR2. Immunity 47:1051–1066. doi:10.1016/j.immuni.2017.11.024 29262348 PMC5772172

[19] Caunt CJ, Keyse SM. 2013. Dual-specificity MAP kinase phosphatases (MKPs): shaping the outcome of MAP kinase signalling. FEBS J 280:489–504. doi:10.1111/j.1742-4658.2012.08716.x 22812510 PMC3594966

[20] Wu T, Hu E, Xu S, Chen M, Guo P, Dai Z, Feng T, Zhou L, Tang W, Zhan L, Fu X, Liu S, Bo X, Yu G. 2021. clusterprofiler 4.0: a universal enrichment tool for interpreting omics data. Innovation (Camb) 2:100141. doi:10.1016/j.xinn.2021.100141 34557778 PMC8454663

[21] Luster AD, Unkeless JC, Ravetch JV. 1985. Gamma-interferon transcriptionally regulates an early-response gene containing homology to platelet proteins. Nature 315:672–676. doi:10.1038/315672a0 3925348

[22] McCowan J, Fercoq F, Kirkwood PM, T’Jonck W, Hegarty LM, Mawer CM, Cunningham R, Mirchandani AS, Hoy A, Humphries DC, Jones G-R, Hansen CG, Hirani N, Jenkins SJ, Henri S, Malissen B, Walmsley SR, Dockrell DH, Saunders PTK, Carlin LM, Bain CC. 2021. The transcription factor EGR2 is indispensable for tissue-specific imprinting of alveolar macrophages in health and tissue repair. Sci Immunol 6:eabj2132. doi:10.1126/sciimmunol.abj2132 34797692 PMC7612216

[23] Pham TH, Benner C, Lichtinger M, Schwarzfischer L, Hu Y, Andreesen R, Chen W, Rehli M. 2012. Dynamic epigenetic enhancer signatures reveal key transcription factors associated with monocytic differentiation states. Blood 119:e161–71. doi:10.1182/blood-2012-01-402453 22550342

[24] Nakamura T, Datta R, Kharbanda S, Kufe D. 1991. Regulation of jun and fos gene expression in human monocytes by the macrophage colony-stimulating factor. Cell Growth Differ 2:267–272. 1712226

[25] Yu H, Maurer F, Medcalf RL. 2002. Plasminogen activator inhibitor type 2: a regulator of monocyte proliferation and differentiation. Blood 99:2810–2818. doi:10.1182/blood.v99.8.2810 11929770

[26] Okamura A, Iwata N, Nagata A, Tamekane A, Shimoyama M, Gomyo H, Yakushijin K, Urahama N, Hamaguchi M, Fukui C, Chihara K, Ito M, Matsui T. 2004. Involvement of casein kinase Iepsilon in cytokine-induced granulocytic differentiation. Blood 103:2997–3004. doi:10.1182/blood-2003-08-2768 15070676

[27] Wynn TA. 2015. Type 2 cytokines: mechanisms and therapeutic strategies. Nat Rev Immunol 15:271–282. doi:10.1038/nri3831 25882242

[28] Uchida H, Kondo A, Yoshimura Y, Mazaki Y, Sabe H. 2001. PAG3/Papalpha/KIAA0400, a GTPase-activating protein for ADP-ribosylation factor (ARF), regulates ARF6 in Fcgamma receptor-mediated phagocytosis of macrophages. J Exp Med 193:955–966. doi:10.1084/jem.193.8.955 11304556 PMC2193405

[29] Nagaraju K, Rawat R, Veszelovszky E, Thapliyal R, Kesari A, Sparks S, Raben N, Plotz P, Hoffman EP. 2008. Dysferlin deficiency enhances monocyte phagocytosis: a model for the inflammatory onset of limb-girdle muscular dystrophy 2B. Am J Pathol 172:774–785. doi:10.2353/ajpath.2008.070327 18276788 PMC2258254

[30] Foley JH, Walton BL, Aleman MM, O’Byrne AM, Lei V, Harrasser M, Foley KA, Wolberg AS, Conway EM. 2016. Complement activation in arterial and venous thrombosis is mediated by plasmin. EBioMedicine 5:175–182. doi:10.1016/j.ebiom.2016.02.011 27077125 PMC4816834

[31] Lomax KJ, Leto TL, Nunoi H, Gallin JI, Malech HL. 1989. Recombinant 47-kilodalton cytosol factor restores NADPH oxidase in chronic granulomatous disease. Science 245:409–412. doi:10.1126/science.2547247 2547247

[32] Volpp BD, Nauseef WM, Donelson JE, Moser DR, Clark RA. 1989. Cloning of the cDNA and functional expression of the 47-kilodalton cytosolic component of human neutrophil respiratory burst oxidase. Proc Natl Acad Sci U S A 86:7195–7199. doi:10.1073/pnas.86.18.7195 2550933 PMC298023

[33] Cutter DiPiazza AR, Taneja N, Dhakshnamoorthy J, Wheeler D, Holla S, Grewal SIS. 2021. Spreading and epigenetic inheritance of heterochromatin require a critical density of histone H3 lysine 9 tri-methylation. Proc Natl Acad Sci USA 118:e2100699118. doi:10.1073/pnas.2100699118 34035174 PMC8179192

[34] Al-Sady B, Madhani HD, Narlikar GJ. 2013. Division of labor between the chromodomains of HP1 and Suv39 methylase enables coordination of heterochromatin spread. Mol Cell 51:80–91. doi:10.1016/j.molcel.2013.06.013 23849629 PMC3752401

[35] Zhang K, Mosch K, Fischle W, Grewal SIS. 2008. Roles of the Clr4 methyltransferase complex in nucleation, spreading and maintenance of heterochromatin. Nat Struct Mol Biol 15:381–388. doi:10.1038/nsmb.1406 18345014

[36] Canzio D, Chang EY, Shankar S, Kuchenbecker KM, Simon MD, Madhani HD, Narlikar GJ, Al-Sady B. 2011. Chromodomain-mediated oligomerization of HP1 suggests a nucleosome-bridging mechanism for heterochromatin assembly. Mol Cell 41:67–81. doi:10.1016/j.molcel.2010.12.016 21211724 PMC3752404

[37] Kharchenko PV, Alekseyenko AA, Schwartz YB, Minoda A, Riddle NC, Ernst J, Sabo PJ, Larschan E, Gorchakov AA, Gu T. 2011. Comprehensive analysis of the chromatin landscape in Drosophila melanogaster. Nature 471:480–485. doi:10.1038/nature09725 21179089 PMC3109908

[38] Bailey LT, Northall SJ, Schalch T. 2021. Breakers and amplifiers in chromatin circuitry: acetylation and ubiquitination control the heterochromatin machinery. Curr Opin Struct Biol 71:156–163. doi:10.1016/j.sbi.2021.06.012 34303934 PMC8667873

[39] Montavon T, Shukeir N, Erikson G, Engist B, Onishi-Seebacher M, Ryan D, Musa Y, Mittler G, Meyer AG, Genoud C, Jenuwein T. 2021. Complete loss of H3K9 methylation dissolves mouse heterochromatin organization. Nat Commun 12:4359. doi:10.1038/s41467-021-24532-8 34272378 PMC8285382

[40] Noma K Allis CD, Grewal SI. 2001. Transitions in distinct histone H3 methylation patterns at the heterochromatin domain boundaries. Science 293:1150–1155. doi:10.1126/science.1064150 11498594

[41] Kurdistani SK, Tavazoie S, Grunstein M. 2004. Mapping global histone acetylation patterns to gene expression. Cell 117:721–733. doi:10.1016/j.cell.2004.05.023 15186774

[42] Wiles ET, Selker EU. 2017. H3K27 methylation: a promiscuous repressive chromatin mark. Curr Opin Genet Dev 43:31–37. doi:10.1016/j.gde.2016.11.001 27940208 PMC5447479

[43] Kuzmichev A, Nishioka K, Erdjument-Bromage H, Tempst P, Reinberg D. 2002. Histone methyltransferase activity associated with a human multiprotein complex containing the enhancer of zeste protein. Genes Dev 16:2893–2905. doi:10.1101/gad.1035902 12435631 PMC187479

[44] Jones B, Su H, Bhat A, Lei H, Bajko J, Hevi S, Baltus GA, Kadam S, Zhai H, Valdez R, Gonzalo S, Zhang Y, Li E, Chen T. 2008. The histone H3K79 methyltransferase Dot1L is essential for mammalian development and heterochromatin structure. PLoS Genet 4:e1000190. doi:10.1371/journal.pgen.1000190 18787701 PMC2527135

[45] Wu H, Chen X, Xiong J, Li Y, Li H, Ding X, Liu S, Chen S, Gao S, Zhu B. 2011. Histone methyltransferase G9a contributes to H3K27 methylation in vivo. Cell Res 21:365–367. doi:10.1038/cr.2010.157 21079650 PMC3193445

[46] Shinkai Y, Tachibana M. 2011. H3K9 methyltransferase G9a and the related molecule GLP. Genes Dev 25:781–788. doi:10.1101/gad.2027411 21498567 PMC3078703

[47] Tachibana M, Ueda J, Fukuda M, Takeda N, Ohta T, Iwanari H, Sakihama T, Kodama T, Hamakubo T, Shinkai Y. 2005. Histone methyltransferases G9a and GLP form heteromeric complexes and are both crucial for methylation of euchromatin at H3-K9. Genes Dev 19:815–826. doi:10.1101/gad.1284005 15774718 PMC1074319

[48] Hume DA. 2008. Differentiation and heterogeneity in the mononuclear phagocyte system. Mucosal Immunol 1:432–441. doi:10.1038/mi.2008.36 19079210

[49] Jakubzick CV, Randolph GJ, Henson PM. 2017. Monocyte differentiation and antigen-presenting functions. Nat Rev Immunol 17:349–362. doi:10.1038/nri.2017.28 28436425

[50] Nakayama J, Rice JC, Strahl BD, Allis CD, Grewal SI. 2001. Role of histone H3 lysine 9 methylation in epigenetic control of heterochromatin assembly. Science 292:110–113. doi:10.1126/science.1060118 11283354

[51] Stock JK, Giadrossi S, Casanova M, Brookes E, Vidal M, Koseki H, Brockdorff N, Fisher AG, Pombo A. 2007. Ring1-mediated ubiquitination of H2A restrains poised RNA polymerase II at bivalent genes in mouse ES cells. Nat Cell Biol 9:1428–1435. doi:10.1038/ncb1663 18037880

[52] Rivera C, Gurard-Levin ZA, Almouzni G, Loyola A. 2014. Histone lysine methylation and chromatin replication. Biochim Biophys Acta 1839:1433–1439. doi:10.1016/j.bbagrm.2014.03.009 24686120

[53] Loyola A, Tagami H, Bonaldi T, Roche D, Quivy JP, Imhof A, Nakatani Y, Dent SYR, Almouzni G. 2009. The HP1alpha-CAF1-SetDB1-containing complex provides H3K9me1 for Suv39-mediated K9me3 in pericentric heterochromatin. EMBO Rep 10:769–775. doi:10.1038/embor.2009.90 19498464 PMC2727428

[54] Nicetto D, Zaret KS. 2019. Role of H3K9me3 heterochromatin in cell identity establishment and maintenance. Curr Opin Genet Dev 55:1–10. doi:10.1016/j.gde.2019.04.013 31103921 PMC6759373

[55] Ecker J, Liebisch G, Englmaier M, Grandl M, Robenek H, Schmitz G. 2010. Induction of fatty acid synthesis is a key requirement for phagocytic differentiation of human monocytes. Proc Natl Acad Sci USA 107:7817–7822. doi:10.1073/pnas.0912059107 20385828 PMC2867858

[56] Zeng L, Li T, Xu DC, Liu J, Mao G, Cui MZ, Fu X, Xu X. 2012. Death receptor 6 induces apoptosis not through type I or type II pathways, but via a unique mitochondria-dependent pathway by interacting with Bax protein. J Biol Chem 287:29125–29133. doi:10.1074/jbc.M112.362038 22761420 PMC3436565

[57] Minakawa T, Kanki Y, Nakamura K, Yamashita JK. 2020. Protein kinase A accelerates the rate of early stage differentiation of pluripotent stem cells. Biochem Biophys Res Commun 524:57–63. doi:10.1016/j.bbrc.2019.12.098 31980180

[58] Ha CH, Kim JY, Zhao J, Wang W, Jhun BS, Wong C, Jin ZG. 2010. PKA phosphorylates histone deacetylase 5 and prevents its nuclear export, leading to the inhibition of gene transcription and cardiomyocyte hypertrophy. Proc Natl Acad Sci U S A 107:15467–15472. doi:10.1073/pnas.1000462107 20716686 PMC2932618

[59] Du M, Perry RL, Nowacki NB, Gordon JW, Salma J, Zhao J, Aziz A, Chan J, Siu KW, McDermott JC. 2008. Protein kinase A represses skeletal myogenesis by targeting myocyte enhancer factor 2D. Mol Cell Biol 28:2952–2970. doi:10.1128/MCB.00248-08 18299387 PMC2293079

[60] Novák J, Fabrik I, Linhartová I, Link M, Černý O, Stulík J, Šebo P. 2017. Phosphoproteomics of cAMP signaling of Bordetella adenylate cyclase toxin in mouse dendritic cells. Sci Rep 7:16298. doi:10.1038/s41598-017-14501-x 29176673 PMC5701129

[61] Gantt SL, Gattis SG, Fierke CA. 2006. Catalytic activity and inhibition of human histone deacetylase 8 is dependent on the identity of the active site metal ion. Biochemistry 45:6170–6178. doi:10.1021/bi060212u 16681389

[62] Bottomley MJ, Lo Surdo P, Di Giovine P, Cirillo A, Scarpelli R, Ferrigno F, Jones P, Neddermann P, De Francesco R, Steinkühler C, Gallinari P, Carfí A. 2008. Structural and functional analysis of the human HDAC4 catalytic domain reveals a regulatory structural zinc-binding domain. J Biol Chem 283:26694–26704. doi:10.1074/jbc.M803514200 18614528 PMC3258910

[63] Finnin MS, Donigian JR, Cohen A, Richon VM, Rifkind RA, Marks PA, Breslow R, Pavletich NP. 1999. Structures of a histone deacetylase homologue bound to the TSA and SAHA inhibitors. Nature 401:188–193. doi:10.1038/43710 10490031

[64] Glozak MA, Sengupta N, Zhang X, Seto E. 2005. Acetylation and deacetylation of non-histone proteins. Gene 363:15–23. doi:10.1016/j.gene.2005.09.010 16289629

[65] Yao YL, Yang WM, Seto E. 2001. Regulation of transcription factor YY1 by acetylation and deacetylation. Mol Cell Biol 21:5979–5991. doi:10.1128/MCB.21.17.5979-5991.2001 11486036 PMC87316

[66] Cerny O, Kamanova J, Masin J, Bibova I, Skopova K, Sebo P. 2015. Bordetella pertussis adenylate cyclase toxin blocks induction of bactericidal nitric oxide in macrophages through cAMP-dependent activation of the SHP-1 phosphatase. J Immunol 194:4901–4913. doi:10.4049/jimmunol.1402941 25876760

[67] Zaman G, Sunters A, Galea GL, Javaheri B, Saxon LK, Moustafa A, Armstrong VJ, Price JS, Lanyon LE. 2012. Loading-related regulation of transcription factor EGR2/Krox-20 in bone cells is ERK1/2 protein-mediated and prostaglandin, Wnt signaling pathway-, and insulin-like growth factor-I axis-dependent. J Biol Chem 287:3946–3962. doi:10.1074/jbc.M111.252742 22049075 PMC3281728

[68] Sheng M, McFadden G, Greenberg ME. 1990. Membrane depolarization and calcium induce c-fos transcription via phosphorylation of transcription factor CREB. Neuron 4:571–582. doi:10.1016/0896-6273(90)90115-v 2157471

[69] Gaynor R, Simon K, Koeffler P. 1991. Expression of c-jun during macrophage differentiation of HL-60 cells. Blood 77:2618–2623. 1904283

[70] Shepherd MC, Baillie GS, Stirling DI, Houslay MD. 2004. Remodelling of the PDE4 cAMP phosphodiesterase isoform profile upon monocyte-macrophage differentiation of human U937 cells. Br J Pharmacol 142:339–351. doi:10.1038/sj.bjp.0705770 15066910 PMC1574950

[71] Ahmad JN, Sebo P. 2024. cAMP signaling of Bordetella adenylate cyclase toxin blocks M-CSF triggered upregulation of iron acquisition receptors on differentiating CD14^+^ monocytes. mSphere 9:e00407-24. doi:10.1128/msphere.00407-24 39078132 PMC11351043

[72] Holubova J, Stanek O, Juhasz A, Hamidou Soumana I, Makovicky P, Sebo P. 2022. The Fim and FhaB adhesins play a crucial role in nasal cavity infection and Bordetella pertussis transmission in a novel mouse catarrhal infection model. PLoS Pathog 18:e1010402. doi:10.1371/journal.ppat.1010402 35395059 PMC9020735

[73] Franken KL, Hiemstra HS, van Meijgaarden KE, Subronto Y, den Hartigh J, Ottenhoff TH, Drijfhout JW. 2000. Purification of his-tagged proteins by immobilized chelate affinity chromatography: the benefits from the use of organic solvent. Protein Expr Purif 18:95–99. doi:10.1006/prep.1999.1162 10648174

[74] Di Tommaso P, Chatzou M, Floden EW, Barja PP, Palumbo E, Notredame C. 2017. Nextflow enables reproducible computational workflows. Nat Biotechnol 35:316–319. doi:10.1038/nbt.3820 28398311

[75] Love MI, Huber W, Anders S. 2014. Moderated estimation of fold change and dispersion for RNA-seq data with DESeq2. Genome Biol 15:550. doi:10.1186/s13059-014-0550-8 25516281 PMC4302049

[76] Zhu A, Ibrahim JG, Love MI. 2019. Heavy-tailed prior distributions for sequence count data: removing the noise and preserving large differences. Bioinformatics 35:2084–2092. doi:10.1093/bioinformatics/bty895 30395178 PMC6581436

[77] Korotkevich G, Sukhov V, Budin N, Shpak B, Artyomov MN, Sergushichev A. 2021. Fast gene set enrichment analysis. bioRxiv. doi:10.1101/060012:060012

