# Supplementary figures and images for: Bordetella adenylate cyclase toxin elicits chromatin remodeling and transcriptional reprogramming that blocks differentiation of monocytes into macrophages
Source: mBio. 2025 Mar 19;16(4):e00138-25. doi: 10.1128/mbio.00138-25 (PMC11980580; doi:10.1128/mbio.00138-25)

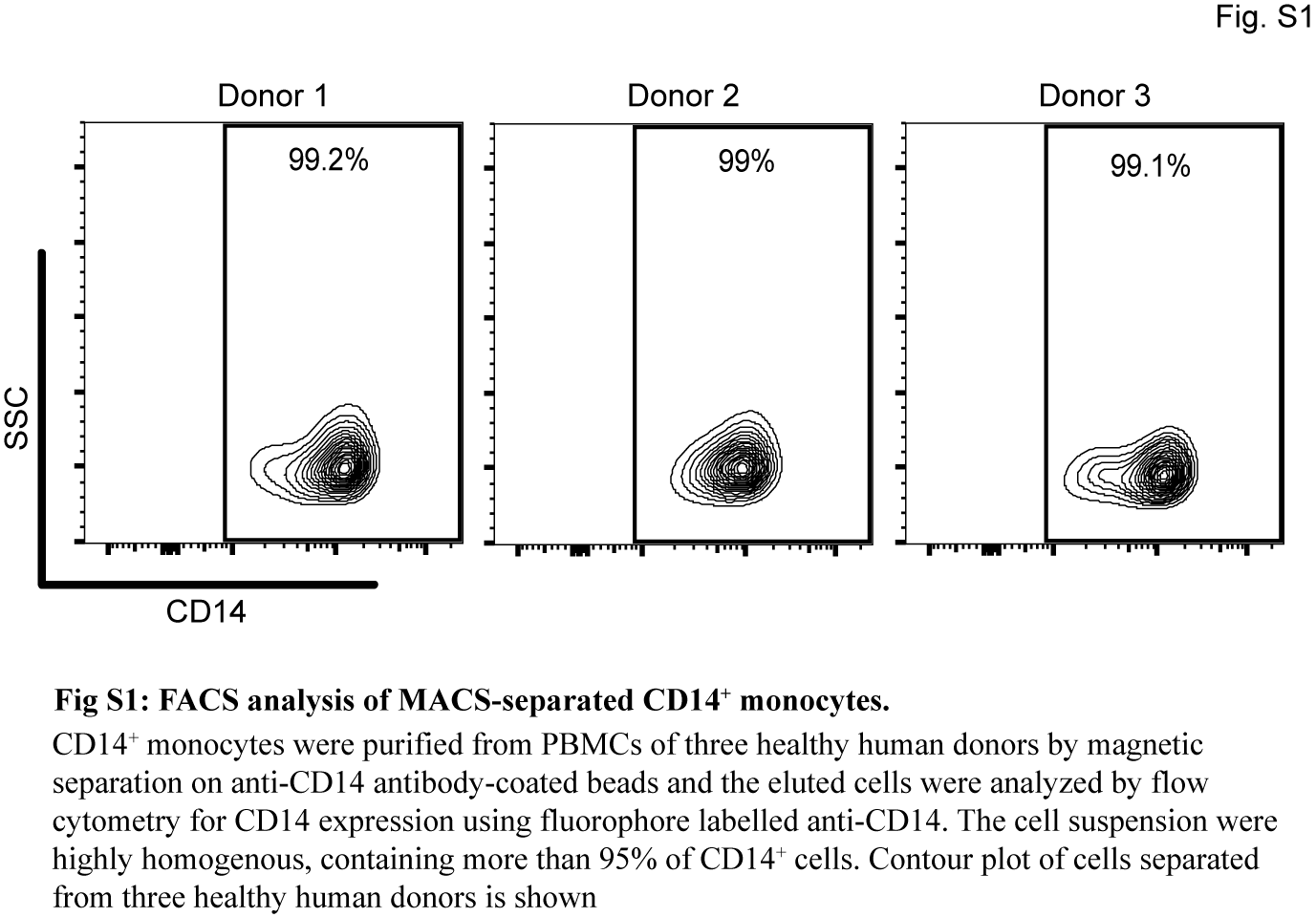

Supplement: Fig. S1 — FACS analysis of purified CD14+ monocytes. [file mbio.00138-25-s0001.tif]

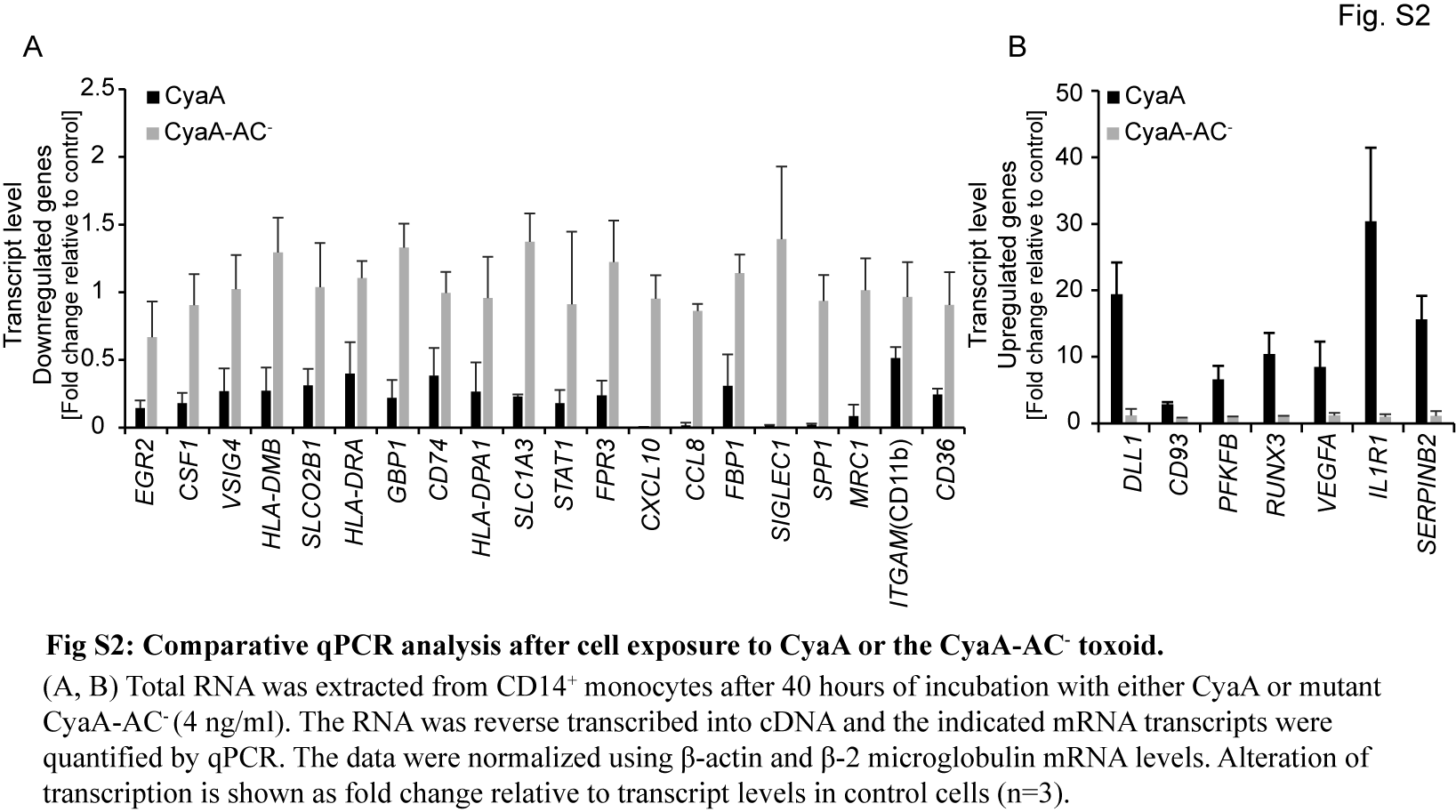

Supplement: Fig. S2 — Comparative qPCR analysis after cell exposure to CyaA or the CyaA-AC- toxoid. [file mbio.00138-25-s0002.tif]

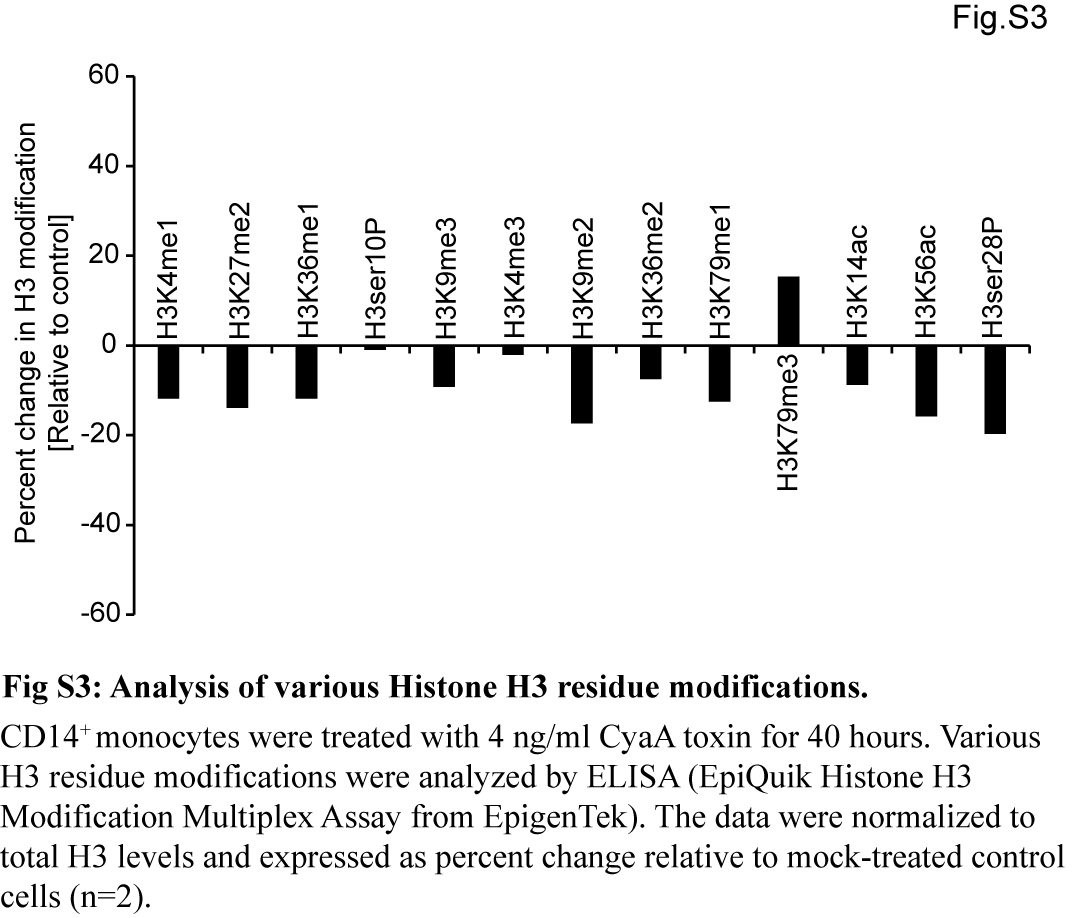

Supplement: Fig. S3 — Analysis of various histone H3 residue modifications. [file mbio.00138-25-s0003.tif]
