# Supplementary material for: Bordetella adenylate cyclase toxin elicits chromatin remodeling and transcriptional reprogramming that blocks differentiation of monocytes into macrophages
Source: mBio. 2025 Mar 19;16(4):e00138-25. doi: 10.1128/mbio.00138-25 (PMC11980580; doi:10.1128/mbio.00138-25)
Supplement: Table S1 — List of all DEG. [file mbio.00138-25-s0004.docx]

**Supplementary Table S1**

**Downregulated genes**

|  | Gene Ensemble ID | Gene name | Base Mean | log2FoldChange | Fold Change | p-adj |
| --- | --- | --- | --- | --- | --- | --- |
|  | ENSG00000169245 | CXCL10 | 106.49 | -9.210 | 0.002 | 2.40E-09 |
|  | ENSG00000011201 | ANOS1 | 62.61 | -8.284 | 0.003 | 1.91E-09 |
|  | ENSG00000135472 | FAIM2 | 24.79 | -8.074 | 0.004 | 3.65E-06 |
|  | ENSG00000106366 | SERPINE1 | 291.99 | -7.306 | 0.006 | 3.35E-35 |
|  | ENSG00000041982 | TNC | 11.91 | -7.016 | 0.008 | 8.81E-04 |
|  | ENSG00000237372 | ENSG00000237372 | 11.82 | -6.999 | 0.008 | 0.00192 |
|  | ENSG00000138755 | CXCL9 | 37.88 | -6.813 | 0.009 | 3.37E-05 |
|  | ENSG00000106178 | CCL24 | 8078.37 | -6.653 | 0.010 | 1.38E-133 |
|  | ENSG00000108700 | CCL8 | 314.63 | -6.628 | 0.010 | 1.01E-31 |
|  | ENSG00000213886 | UBD | 8.63 | -6.544 | 0.011 | 0.013702 |
|  | ENSG00000100079 | LGALS2 | 16.67 | -6.518 | 0.011 | 0.003661 |
|  | ENSG00000115648 | MLPH | 8.01 | -6.389 | 0.012 | 0.029817 |
|  | ENSG00000260314 | MRC1 | 2211.01 | -6.359 | 0.012 | 1.83E-91 |
|  | ENSG00000076864 | RAP1GAP | 15.30 | -6.327 | 0.012 | 0.004849 |
|  | ENSG00000130222 | GADD45G | 82.67 | -6.260 | 0.013 | 3.11E-10 |
|  | ENSG00000165140 | FBP1 | 1233.77 | -6.247 | 0.013 | 8.09E-92 |
|  | ENSG00000181374 | CCL13 | 142.36 | -6.130 | 0.014 | 1.72E-16 |
|  | ENSG00000100336 | APOL4 | 87.67 | -6.120 | 0.014 | 1.16E-12 |
|  | ENSG00000076716 | GPC4 | 6.07 | -6.047 | 0.015 | 0.048019 |
|  | ENSG00000198785 | GRIN3A | 10.47 | -5.847 | 0.017 | 0.026568 |
|  | ENSG00000174307 | PHLDA3 | 15.60 | -5.807 | 0.018 | 0.029331 |
|  | ENSG00000132514 | CLEC10A | 881.96 | -5.789 | 0.018 | 1.19E-41 |
|  | ENSG00000158270 | COLEC12 | 18.01 | -5.708 | 0.019 | 0.004469 |
|  | ENSG00000158485 | CD1B | 33.04 | -5.617 | 0.020 | 9.64E-06 |
|  | ENSG00000010030 | ETV7 | 124.99 | -5.561 | 0.021 | 3.41E-20 |
|  | ENSG00000225492 | GBP1P1 | 23.69 | -5.548 | 0.021 | 0.001133 |
|  | ENSG00000118785 | SPP1 | 74.41 | -5.532 | 0.022 | 1.13E-08 |
|  | ENSG00000290525 | GBP1P1 | 21.77 | -5.405 | 0.024 | 0.001157 |
|  | ENSG00000158488 | CD1E | 67.96 | -5.372 | 0.024 | 3.75E-10 |
|  | ENSG00000108702 | CCL1 | 68.51 | -5.350 | 0.025 | 2.80E-11 |
|  | ENSG00000137203 | TFAP2A | 34.51 | -5.287 | 0.026 | 2.28E-05 |
|  | ENSG00000184313 | MROH7 | 19.07 | -5.274 | 0.026 | 0.003301 |
|  | ENSG00000091831 | ESR1 | 18.15 | -5.239 | 0.026 | 0.002294 |
|  | ENSG00000158163 | DZIP1L | 22.55 | -5.233 | 0.027 | 0.001265 |
|  | ENSG00000187474 | FPR3 | 1678.89 | -5.222 | 0.027 | 4.51E-46 |
|  | ENSG00000164935 | DCSTAMP | 68.95 | -5.104 | 0.029 | 4.84E-12 |
|  | ENSG00000122877 | EGR2 | 1007.088 | -5.058 | 0.030 | 9.92E-72 |
|  | ENSG00000184371 | CSF1 | 695.98 | -5.021 | 0.031 | 1.21E-48 |
|  | ENSG00000185245 | GP1BA | 37.85 | -4.976 | 0.032 | 2.33E-06 |
|  | ENSG00000113302 | IL12B | 49.14 | -4.847 | 0.035 | 1.84E-07 |
|  | ENSG00000163624 | CDS1 | 19.57 | -4.825 | 0.035 | 0.001533 |
|  | ENSG00000275385 | CCL18 | 50.57 | -4.819 | 0.035 | 1.46E-08 |
|  | ENSG00000155659 | VSIG4 | 647.45 | -4.811 | 0.036 | 1.10E-49 |
|  | ENSG00000172322 | CLEC12A | 278.20 | -4.808 | 0.036 | 8.86E-41 |
|  | ENSG00000127954 | STEAP4 | 300.55 | -4.803 | 0.036 | 2.35E-23 |
|  | ENSG00000242574 | HLA.DMB | 1861.71 | -4.742 | 0.037 | 2.79E-35 |
|  | ENSG00000117594 | HSD11B1 | 106.83 | -4.716 | 0.038 | 2.95E-13 |
|  | ENSG00000111537 | IFNG | 72.14 | -4.690 | 0.039 | 2.90E-10 |
|  | ENSG00000226822 | LINC02785 | 14.80 | -4.599 | 0.041 | 0.035312 |
|  | ENSG00000205846 | CLEC6A | 132.73 | -4.584 | 0.042 | 3.75E-20 |
|  | ENSG00000185433 | LINC00158 | 44.86 | -4.557 | 0.042 | 1.33E-06 |
|  | ENSG00000262097 | LINC02185 | 14.59 | -4.547 | 0.043 | 0.01132 |
|  | ENSG00000085265 | FCN1 | 1387.53 | -4.531 | 0.043 | 1.45E-68 |
|  | ENSG00000289053 | ENSG00000289053 | 19.97 | -4.526 | 0.043 | 0.00212 |
|  | ENSG00000166922 | SCG5 | 24.19 | -4.518 | 0.044 | 9.65E-04 |
|  | ENSG00000124479 | NDP | 71.44 | -4.442 | 0.046 | 1.01E-09 |
|  | ENSG00000262406 | MMP12 | 120.38 | -4.436 | 0.046 | 8.65E-19 |
|  | ENSG00000149635 | OCSTAMP | 161.88 | -4.405 | 0.047 | 1.11E-23 |
|  | ENSG00000149131 | SERPING1 | 246.02 | -4.323 | 0.050 | 4.02E-28 |
|  | ENSG00000088827 | SIGLEC1 | 43.69 | -4.321 | 0.050 | 1.07E-05 |
|  | ENSG00000169896 | ITGAM | 1301.92 | -4.261 | 0.052 | 7.87E-23 |
|  | ENSG00000117228 | GBP1 | 2936.55 | -4.234 | 0.053 | 2.98E-40 |
|  | ENSG00000143184 | XCL1 | 16.30 | -4.216 | 0.054 | 0.018426 |
|  | ENSG00000174945 | AMZ1 | 63.22 | -4.190 | 0.055 | 1.23E-07 |
|  | ENSG00000169116 | PARM1 | 24.93 | -4.117 | 0.058 | 0.027739 |
|  | ENSG00000168062 | BATF2 | 278.42 | -4.085 | 0.059 | 2.68E-19 |
|  | ENSG00000115457 | IGFBP2 | 15.60 | -4.054 | 0.060 | 0.045921 |
|  | ENSG00000050344 | NFE2L3 | 367.09 | -4.047 | 0.061 | 2.55E-26 |
|  | ENSG00000166689 | PLEKHA7 | 65.23 | -4.013 | 0.062 | 8.61E-08 |
|  | ENSG00000148180 | GSN | 4351.69 | -3.980 | 0.063 | 1.02E-38 |
|  | ENSG00000260135 | MMP2.AS1 | 38.82 | -3.895 | 0.067 | 3.84E-04 |
|  | ENSG00000148483 | TMEM236 | 21.24 | -3.887 | 0.068 | 0.00694 |
|  | ENSG00000137673 | MMP7 | 420.10 | -3.869 | 0.068 | 3.95E-20 |
|  | ENSG00000011028 | MRC2 | 24.11 | -3.845 | 0.070 | 0.010153 |
|  | ENSG00000112799 | LY86 | 187.93 | -3.839 | 0.070 | 2.80E-11 |
|  | ENSG00000100490 | CDKL1 | 37.32 | -3.801 | 0.072 | 4.36E-05 |
|  | ENSG00000169255 | B3GALNT1 | 36.24 | -3.736 | 0.075 | 8.08E-04 |
|  | ENSG00000290878 | HLA.DRB6 | 269.79 | -3.708 | 0.077 | 2.99E-14 |
|  | ENSG00000256268 | LINC02454 | 19.28 | -3.687 | 0.078 | 0.04495 |
|  | ENSG00000120885 | CLU | 584.91 | -3.658 | 0.079 | 6.32E-16 |
|  | ENSG00000258227 | CLEC5A | 3746.05 | -3.656 | 0.079 | 1.05E-18 |
|  | ENSG00000147570 | DNAJC5B | 49.39 | -3.603 | 0.082 | 6.65E-06 |
|  | ENSG00000179344 | HLA.DQB1 | 393.99 | -3.583 | 0.083 | 4.43E-21 |
|  | ENSG00000291144 | ENSG00000291144 | 30.76 | -3.547 | 0.086 | 0.004648 |
|  | ENSG00000251301 | LINC02384 | 141.96 | -3.510 | 0.088 | 1.13E-08 |
|  | ENSG00000170956 | CEACAM3 | 77.67 | -3.501 | 0.088 | 4.19E-08 |
|  | ENSG00000072694 | FCGR2B | 5245.15 | -3.486 | 0.089 | 1.77E-30 |
|  | ENSG00000204287 | HLA.DRA | 12015.06 | -3.476 | 0.090 | 2.47E-39 |
|  | ENSG00000198502 | HLA.DRB5 | 530.50 | -3.469 | 0.090 | 3.75E-21 |
|  | ENSG00000168961 | LGALS9 | 718.98 | -3.467 | 0.090 | 4.43E-32 |
|  | ENSG00000162654 | GBP4 | 980.21 | -3.465 | 0.091 | 1.61E-20 |
|  | ENSG00000100311 | PDGFB | 77.11 | -3.442 | 0.092 | 1.57E-08 |
|  | ENSG00000109321 | AREG | 28.31 | -3.420 | 0.093 | 0.025235 |
|  | ENSG00000204161 | TMEM273 | 109.04 | -3.395 | 0.095 | 5.27E-07 |
|  | ENSG00000118292 | C1orf54 | 28.03 | -3.368 | 0.097 | 0.007814 |
|  | ENSG00000257335 | MGAM | 75.56 | -3.354 | 0.098 | 6.61E-06 |
|  | ENSG00000231389 | HLA.DPA1 | 4156.98 | -3.333 | 0.099 | 4.13E-23 |
|  | ENSG00000255750 | ENSG00000255750 | 140.00 | -3.326 | 0.100 | 5.62E-11 |
|  | ENSG00000065833 | ME1 | 27.11 | -3.295 | 0.102 | 0.010303 |
|  | ENSG00000204257 | HLA.DMA | 1322.10 | -3.284 | 0.103 | 1.18E-25 |
|  | ENSG00000137491 | SLCO2B1 | 248.88 | -3.276 | 0.103 | 3.71E-12 |
|  | ENSG00000223865 | HLA.DPB1 | 2211.18 | -3.268 | 0.104 | 3.93E-29 |
|  | ENSG00000135218 | CD36 | 202.57 | -3.266 | 0.104 | 1.85E-10 |
|  | ENSG00000150337 | FCGR1A | 1059.09 | -3.256 | 0.105 | 1.53E-08 |
|  | ENSG00000087589 | CASS4 | 142.02 | -3.241 | 0.106 | 2.72E-09 |
|  | ENSG00000163121 | NEURL3 | 22.45 | -3.234 | 0.106 | 0.034432 |
|  | ENSG00000172724 | CCL19 | 164.60 | -3.231 | 0.107 | 8.98E-11 |
|  | ENSG00000173391 | OLR1 | 681.66 | -3.225 | 0.107 | 3.91E-20 |
|  | ENSG00000179583 | CIITA | 1491.30 | -3.225 | 0.107 | 5.67E-12 |
|  | ENSG00000118242 | MREG | 274.40 | -3.220 | 0.107 | 3.57E-13 |
|  | ENSG00000196126 | HLA.DRB1 | 4410.67 | -3.218 | 0.107 | 1.48E-21 |
|  | ENSG00000165178 | NCF1C | 2786.22 | -3.211 | 0.108 | 9.46E-09 |
|  | ENSG00000285103 | ENSG00000285103 | 70.47 | -3.204 | 0.109 | 4.63E-06 |
|  | ENSG00000186827 | TNFRSF4 | 80.74 | -3.203 | 0.109 | 0.015682 |
|  | ENSG00000133321 | PLAAT4 | 212.34 | -3.189 | 0.110 | 9.53E-06 |
|  | ENSG00000225217 | HSPA7 | 694.42 | -3.181 | 0.110 | 2.50E-08 |
|  | ENSG00000007264 | MATK | 102.63 | -3.178 | 0.110 | 9.82E-07 |
|  | ENSG00000154451 | GBP5 | 1237.31 | -3.175 | 0.111 | 1.19E-11 |
|  | ENSG00000115415 | STAT1 | 7453.53 | -3.152 | 0.113 | 1.98E-37 |
|  | ENSG00000135476 | ESPL1 | 46.93 | -3.140 | 0.113 | 0.006638 |
|  | ENSG00000256234 | ITPR2.AS1 | 86.38 | -3.131 | 0.114 | 5.08E-07 |
|  | ENSG00000101000 | PROCR | 374.02 | -3.128 | 0.114 | 9.30E-10 |
|  | ENSG00000146592 | CREB5 | 332.71 | -3.127 | 0.114 | 3.20E-13 |
|  | ENSG00000227825 | SLC9A7P1 | 130.82 | -3.119 | 0.115 | 8.02E-04 |
|  | ENSG00000271503 | CCL5 | 800.71 | -3.109 | 0.116 | 3.23E-14 |
|  | ENSG00000126262 | FFAR2 | 450.80 | -3.094 | 0.117 | 6.01E-05 |
|  | ENSG00000111344 | RASAL1 | 47.40 | -3.056 | 0.120 | 0.008669 |
|  | ENSG00000137834 | SMAD6 | 32.35 | -3.036 | 0.122 | 0.028421 |
|  | ENSG00000139970 | RTN1 | 430.04 | -3.017 | 0.124 | 1.60E-18 |
|  | ENSG00000166670 | MMP10 | 110.77 | -2.994 | 0.126 | 1.92E-05 |
|  | ENSG00000136235 | GPNMB | 351.13 | -2.990 | 0.126 | 4.34E-09 |
|  | ENSG00000244682 | FCGR2C | 586.25 | -2.987 | 0.126 | 8.64E-06 |
|  | ENSG00000019582 | CD74 | 19268.97 | -2.982 | 0.127 | 1.06E-32 |
|  | ENSG00000152766 | ANKRD22 | 1151.37 | -2.981 | 0.127 | 4.43E-10 |
|  | ENSG00000102760 | RGCC | 203.04 | -2.979 | 0.127 | 1.01E-05 |
|  | ENSG00000162390 | ACOT11 | 71.21 | -2.978 | 0.127 | 0.005922 |
|  | ENSG00000133106 | EPSTI1 | 545.98 | -2.964 | 0.128 | 1.59E-07 |
|  | ENSG00000187037 | GPR141 | 97.87 | -2.953 | 0.129 | 2.29E-05 |
|  | ENSG00000119917 | IFIT3 | 462.26 | -2.938 | 0.130 | 8.04E-09 |
|  | ENSG00000117226 | GBP3 | 259.05 | -2.899 | 0.134 | 6.54E-12 |
|  | ENSG00000232629 | HLA.DQB2 | 94.06 | -2.896 | 0.134 | 2.39E-05 |
|  | ENSG00000139410 | SDSL | 137.24 | -2.894 | 0.135 | 3.32E-05 |
|  | ENSG00000166278 | C2 | 170.31 | -2.885 | 0.135 | 2.49E-08 |
|  | ENSG00000198520 | ARMH1 | 76.63 | -2.874 | 0.136 | 0.015599 |
|  | ENSG00000122641 | INHBA | 2098.53 | -2.866 | 0.137 | 4.50E-26 |
|  | ENSG00000065923 | SLC9A7 | 133.91 | -2.859 | 0.138 | 8.64E-06 |
|  | ENSG00000148459 | PDSS1 | 182.76 | -2.857 | 0.138 | 3.68E-08 |
|  | ENSG00000240065 | PSMB9 | 1363.63 | -2.847 | 0.139 | 1.44E-07 |
|  | ENSG00000168899 | VAMP5 | 401.97 | -2.841 | 0.140 | 8.82E-04 |
|  | ENSG00000140511 | HAPLN3 | 410.07 | -2.840 | 0.140 | 3.30E-10 |
|  | ENSG00000117009 | KMO | 719.50 | -2.832 | 0.140 | 4.20E-16 |
|  | ENSG00000002549 | LAP3 | 3448.14 | -2.830 | 0.141 | 2.07E-22 |
|  | ENSG00000102524 | TNFSF13B | 416.73 | -2.797 | 0.144 | 4.48E-08 |
|  | ENSG00000119922 | IFIT2 | 104.54 | -2.787 | 0.145 | 1.15E-04 |
|  | ENSG00000274276 | ENSG00000274276 | 51.69 | -2.766 | 0.147 | 0.006425 |
|  | ENSG00000127951 | FGL2 | 1182.00 | -2.760 | 0.148 | 1.31E-04 |
|  | ENSG00000234883 | MIR155HG | 380.19 | -2.745 | 0.149 | 7.23E-08 |
|  | ENSG00000265531 | FCGR1CP | 103.23 | -2.733 | 0.150 | 0.002306 |
|  | ENSG00000125347 | IRF1 | 5479.08 | -2.701 | 0.154 | 6.61E-16 |
|  | ENSG00000237541 | HLA.DQA2 | 460.44 | -2.694 | 0.155 | 1.85E-12 |
|  | ENSG00000079215 | SLC1A3 | 774.63 | -2.676 | 0.156 | 2.90E-10 |
|  | ENSG00000290838 | NCF1B | 1128.73 | -2.654 | 0.159 | 1.98E-07 |
|  | ENSG00000291135 | FCGR1BP | 264.92 | -2.651 | 0.159 | 3.32E-05 |
|  | ENSG00000102575 | ACP5 | 1842.89 | -2.647 | 0.160 | 9.42E-06 |
|  | ENSG00000089041 | P2RX7 | 1324.03 | -2.643 | 0.160 | 1.69E-12 |
|  | ENSG00000134780 | DAGLA | 145.40 | -2.639 | 0.161 | 2.15E-04 |
|  | ENSG00000136514 | RTP4 | 114.29 | -2.638 | 0.161 | 5.01E-05 |
|  | ENSG00000144681 | STAC | 533.91 | -2.627 | 0.162 | 1.02E-12 |
|  | ENSG00000150637 | CD226 | 384.51 | -2.599 | 0.165 | 5.97E-06 |
|  | ENSG00000148803 | FUOM | 132.49 | -2.598 | 0.165 | 0.005472 |
|  | ENSG00000161929 | SCIMP | 397.72 | -2.596 | 0.165 | 3.64E-05 |
|  | ENSG00000126264 | HCST | 153.70 | -2.595 | 0.165 | 0.003493 |
|  | ENSG00000267120 | ENSG00000267120 | 175.32 | -2.594 | 0.166 | 8.20E-06 |
|  | ENSG00000162645 | GBP2 | 1699.03 | -2.586 | 0.167 | 1.64E-13 |
|  | ENSG00000034510 | TMSB10 | 11745.01 | -2.583 | 0.167 | 1.36E-04 |
|  | ENSG00000106211 | HSPB1 | 318.23 | -2.566 | 0.169 | 3.09E-04 |
|  | ENSG00000162746 | FCRLB | 146.46 | -2.528 | 0.173 | 3.67E-06 |
|  | ENSG00000126709 | IFI6 | 380.67 | -2.519 | 0.174 | 0.015892 |
|  | ENSG00000187098 | MITF | 357.91 | -2.519 | 0.175 | 3.17E-08 |
|  | ENSG00000164440 | TXLNB | 158.29 | -2.516 | 0.175 | 0.003121 |
|  | ENSG00000181634 | TNFSF15 | 109.17 | -2.499 | 0.177 | 0.00212 |
|  | ENSG00000226091 | LINC00937 | 405.40 | -2.481 | 0.179 | 2.04E-06 |
|  | ENSG00000160593 | JAML | 1601.05 | -2.479 | 0.179 | 2.22E-07 |
|  | ENSG00000164509 | IL31RA | 143.33 | -2.456 | 0.182 | 8.43E-06 |
|  | ENSG00000111181 | SLC6A12 | 112.88 | -2.449 | 0.183 | 0.00125 |
|  | ENSG00000158517 | NCF1 | 6104.50 | -2.446 | 0.184 | 1.74E-07 |
|  | ENSG00000152229 | PSTPIP2 | 1904.40 | -2.444 | 0.184 | 6.64E-11 |
|  | ENSG00000115525 | ST3GAL5 | 213.31 | -2.443 | 0.184 | 1.75E-06 |
|  | ENSG00000103855 | CD276 | 222.61 | -2.437 | 0.185 | 2.42E-04 |
|  | ENSG00000071205 | ARHGAP10 | 223.47 | -2.409 | 0.188 | 1.51E-07 |
|  | ENSG00000169413 | RNASE6 | 105.12 | -2.393 | 0.190 | 9.86E-04 |
|  | ENSG00000163702 | IL17RC | 92.58 | -2.391 | 0.191 | 0.001008 |
|  | ENSG00000136059 | VILL | 360.53 | -2.378 | 0.192 | 9.29E-06 |
|  | ENSG00000197471 | SPN | 946.80 | -2.375 | 0.193 | 1.10E-08 |
|  | ENSG00000163563 | MNDA | 294.21 | -2.373 | 0.193 | 8.41E-04 |
|  | ENSG00000179593 | ALOX15B | 218.88 | -2.370 | 0.193 | 1.06E-05 |
|  | ENSG00000126246 | IGFLR1 | 952.54 | -2.367 | 0.194 | 0.001082 |
|  | ENSG00000162882 | HAAO | 63.41 | -2.352 | 0.196 | 0.016296 |
|  | ENSG00000121858 | TNFSF10 | 764.64 | -2.350 | 0.196 | 4.73E-06 |
|  | ENSG00000135069 | PSAT1 | 150.21 | -2.349 | 0.196 | 1.41E-05 |
|  | ENSG00000089127 | OAS1 | 323.76 | -2.343 | 0.197 | 2.37E-05 |
|  | ENSG00000138135 | CH25H | 46.30 | -2.336 | 0.198 | 0.044933 |
|  | ENSG00000172403 | SYNPO2 | 163.69 | -2.321 | 0.200 | 0.014112 |
|  | ENSG00000261040 | WFDC21P | 183.62 | -2.316 | 0.201 | 0.017741 |
|  | ENSG00000182782 | HCAR2 | 343.45 | -2.315 | 0.201 | 2.10E-05 |
|  | ENSG00000085733 | CTTN | 159.21 | -2.309 | 0.202 | 0.002653 |
|  | ENSG00000123095 | BHLHE41 | 1011.30 | -2.308 | 0.202 | 9.89E-04 |
|  | ENSG00000145936 | KCNMB1 | 250.34 | -2.289 | 0.205 | 0.003493 |
|  | ENSG00000121316 | PLBD1 | 246.11 | -2.287 | 0.205 | 1.77E-05 |
|  | ENSG00000139567 | ACVRL1 | 244.98 | -2.286 | 0.205 | 0.005338 |
|  | ENSG00000137496 | IL18BP | 353.52 | -2.282 | 0.206 | 1.76E-06 |
|  | ENSG00000075618 | FSCN1 | 451.79 | -2.266 | 0.208 | 9.39E-06 |
|  | ENSG00000100911 | PSME2 | 1675.18 | -2.258 | 0.209 | 3.69E-06 |
|  | ENSG00000291143 | ENSG00000291143 | 203.01 | -2.254 | 0.210 | 0.003824 |
|  | ENSG00000109861 | CTSC | 2294.29 | -2.238 | 0.212 | 1.55E-08 |
|  | ENSG00000205927 | OLIG2 | 116.16 | -2.230 | 0.213 | 0.019198 |
|  | ENSG00000156587 | UBE2L6 | 1317.48 | -2.218 | 0.215 | 7.88E-07 |
|  | ENSG00000128284 | APOL3 | 1807.49 | -2.210 | 0.216 | 7.35E-10 |
|  | ENSG00000101384 | JAG1 | 120.51 | -2.195 | 0.218 | 0.003859 |
|  | ENSG00000151693 | ASAP2 | 136.13 | -2.157 | 0.224 | 0.005605 |
|  | ENSG00000050405 | LIMA1 | 164.13 | -2.137 | 0.227 | 0.004198 |
|  | ENSG00000177606 | JUN | 918.38 | -2.137 | 0.227 | 1.51E-08 |
|  | ENSG00000168394 | TAP1 | 3708.61 | -2.130 | 0.228 | 3.98E-10 |
|  | ENSG00000102962 | CCL22 | 489.47 | -2.128 | 0.229 | 3.83E-06 |
|  | ENSG00000138119 | MYOF | 2269.48 | -2.127 | 0.229 | 0.006119 |
|  | ENSG00000099377 | HSD3B7 | 165.82 | -2.125 | 0.229 | 0.00742 |
|  | ENSG00000255833 | TIFAB | 178.38 | -2.123 | 0.230 | 0.015919 |
|  | ENSG00000106565 | TMEM176B | 396.82 | -2.121 | 0.230 | 0.004751 |
|  | ENSG00000160883 | HK3 | 1339.06 | -2.118 | 0.230 | 0.033549 |
|  | ENSG00000120162 | MOB3B | 727.92 | -2.110 | 0.232 | 0.001406 |
|  | ENSG00000166347 | CYB5A | 106.85 | -2.109 | 0.232 | 0.034897 |
|  | ENSG00000167600 | CYP2S1 | 188.25 | -2.093 | 0.234 | 7.67E-04 |
|  | ENSG00000138316 | ADAMTS14 | 562.58 | -2.088 | 0.235 | 3.25E-05 |
|  | ENSG00000108691 | CCL2 | 43907.92 | -2.082 | 0.236 | 5.62E-04 |
|  | ENSG00000138080 | EMILIN1 | 242.11 | -2.070 | 0.238 | 0.031883 |
|  | ENSG00000182287 | AP1S2 | 703.29 | -2.068 | 0.238 | 8.08E-07 |
|  | ENSG00000164691 | TAGAP | 510.73 | -2.048 | 0.242 | 2.46E-05 |
|  | ENSG00000101255 | TRIB3 | 462.33 | -2.033 | 0.244 | 0.002302 |
|  | ENSG00000102755 | FLT1 | 730.11 | -2.027 | 0.245 | 0.010271 |
|  | ENSG00000136205 | TNS3 | 1267.77 | -2.021 | 0.246 | 0.009465 |
|  | ENSG00000002933 | TMEM176A | 170.01 | -2.006 | 0.249 | 0.036676 |
|  | ENSG00000243649 | CFB | 1176.09 | -1.991 | 0.251 | 1.08E-07 |
|  | ENSG00000120708 | TGFBI | 1476.67 | -1.990 | 0.252 | 0.030145 |
|  | ENSG00000111729 | CLEC4A | 2409.14 | -1.978 | 0.254 | 8.41E-04 |
|  | ENSG00000187630 | DHRS4L2 | 270.01 | -1.944 | 0.260 | 0.009388 |
|  | ENSG00000205358 | MT1H | 4473.78 | -1.924 | 0.264 | 0.00228 |
|  | ENSG00000196735 | HLA.DQA1 | 1588.48 | -1.917 | 0.265 | 9.69E-05 |
|  | ENSG00000154310 | TNIK | 374.29 | -1.910 | 0.266 | 0.029296 |
|  | ENSG00000108688 | CCL7 | 486.61 | -1.903 | 0.267 | 0.004685 |
|  | ENSG00000160791 | CCR5 | 2736.20 | -1.888 | 0.270 | 0.006726 |
|  | ENSG00000010610 | CD4 | 285.27 | -1.868 | 0.274 | 0.026568 |
|  | ENSG00000146859 | TMEM140 | 193.96 | -1.868 | 0.274 | 0.046036 |
|  | ENSG00000122644 | ARL4A | 365.68 | -1.866 | 0.274 | 0.003824 |
|  | ENSG00000140749 | IGSF6 | 3864.19 | -1.831 | 0.281 | 0.032123 |
|  | ENSG00000138678 | GPAT3 | 208.68 | -1.829 | 0.282 | 0.016835 |
|  | ENSG00000101017 | CD40 | 1622.89 | -1.816 | 0.284 | 2.60E-04 |
|  | ENSG00000120262 | CCDC170 | 213.84 | -1.801 | 0.287 | 0.029574 |
|  | ENSG00000092621 | PHGDH | 562.04 | -1.780 | 0.291 | 0.002633 |
|  | ENSG00000221963 | APOL6 | 2610.80 | -1.770 | 0.293 | 0.003411 |
|  | ENSG00000100342 | APOL1 | 788.55 | -1.763 | 0.295 | 0.006366 |
|  | ENSG00000146376 | ARHGAP18 | 622.43 | -1.752 | 0.297 | 0.036112 |
|  | ENSG00000111275 | ALDH2 | 1467.61 | -1.745 | 0.298 | 0.00135 |
|  | ENSG00000186818 | LILRB4 | 1254.24 | -1.732 | 0.301 | 0.026726 |
|  | ENSG00000074964 | ARHGEF10L | 789.89 | -1.726 | 0.302 | 0.045947 |
|  | ENSG00000100985 | MMP9 | 41949.27 | -1.700 | 0.308 | 3.54E-04 |
|  | ENSG00000138496 | PARP9 | 1382.23 | -1.696 | 0.309 | 0.007125 |
|  | ENSG00000138670 | RASGEF1B | 328.82 | -1.692 | 0.309 | 0.039659 |
|  | ENSG00000122694 | GLIPR2 | 1680.70 | -1.684 | 0.311 | 0.026057 |
|  | ENSG00000086300 | SNX10 | 2399.42 | -1.678 | 0.313 | 0.015892 |
|  | ENSG00000147168 | IL2RG | 1831.55 | -1.602 | 0.329 | 0.003285 |
|  | ENSG00000118515 | SGK1 | 4288.83 | -1.586 | 0.333 | 0.005701 |

**Upregulated Genes**

|  | Gene Ensemble ID | Gene name | Base Mean | log2FoldChange | Fold Change | p-adj |
| --- | --- | --- | --- | --- | --- | --- |
|  | ENSG00000124882 | EREG | 6682.71 | 1.597 | 3.026 | 0.039863 |
|  | ENSG00000129353 | SLC44A2 | 1322.06 | 1.648 | 3.134 | 0.004751 |
|  | ENSG00000113369 | ARRDC3 | 612.83 | 1.654 | 3.147 | 0.032123 |
|  | ENSG00000112394 | SLC16A10 | 486.80 | 1.686 | 3.219 | 0.029574 |
|  | ENSG00000132199 | ENOSF1 | 845.78 | 1.692 | 3.231 | 0.014477 |
|  | ENSG00000135373 | EHF | 507.85 | 1.765 | 3.400 | 0.027248 |
|  | ENSG00000186918 | ZNF395 | 379.90 | 1.779 | 3.432 | 0.010007 |
|  | ENSG00000153395 | LPCAT1 | 2462.56 | 1.781 | 3.438 | 3.72E-05 |
|  | ENSG00000213923 | CSNK1E | 882.59 | 1.859 | 3.626 | 0.004533 |
|  | ENSG00000165474 | GJB2 | 1689.14 | 1.881 | 3.683 | 2.10E-05 |
|  | ENSG00000291109 | CES1P1 | 341.14 | 1.929 | 3.807 | 0.004855 |
|  | ENSG00000147454 | SLC25A37 | 16427.49 | 1.953 | 3.872 | 3.52E-07 |
|  | ENSG00000170525 | PFKFB3 | 30254.35 | 1.960 | 3.890 | 0.001133 |
|  | ENSG00000113448 | PDE4D | 500.39 | 1.964 | 3.901 | 0.001443 |
|  | ENSG00000100600 | LGMN | 918.90 | 1.979 | 3.943 | 8.44E-05 |
|  | ENSG00000205755 | CRLF2 | 228.08 | 2.008 | 4.024 | 0.005033 |
|  | ENSG00000105976 | MET | 1529.23 | 2.025 | 4.071 | 6.09E-04 |
|  | ENSG00000112715 | VEGFA | 3102.11 | 2.067 | 4.191 | 7.53E-04 |
|  | ENSG00000197256 | KANK2 | 166.07 | 2.097 | 4.279 | 0.01407 |
|  | ENSG00000136634 | IL10 | 935.78 | 2.140 | 4.407 | 3.09E-04 |
|  | ENSG00000198719 | DLL1 | 998.80 | 2.160 | 4.468 | 1.46E-08 |
|  | ENSG00000104635 | SLC39A14 | 274.55 | 2.169 | 4.497 | 0.002023 |
|  | ENSG00000128578 | STRIP2 | 358.94 | 2.190 | 4.565 | 5.33E-04 |
|  | ENSG00000204020 | LIPN | 64.66 | 2.210 | 4.627 | 0.034015 |
|  | ENSG00000115604 | IL18R1 | 108.86 | 2.227 | 4.682 | 0.034015 |
|  | ENSG00000159496 | RGL4 | 157.38 | 2.248 | 4.751 | 0.004106 |
|  | ENSG00000093134 | VNN3P | 2189.45 | 2.263 | 4.801 | 2.08E-12 |
|  | ENSG00000127418 | FGFRL1 | 409.74 | 2.276 | 4.842 | 3.26E-08 |
|  | ENSG00000118596 | SLC16A7 | 2095.31 | 2.288 | 4.885 | 2.05E-09 |
|  | ENSG00000196460 | RFX8 | 192.52 | 2.337 | 5.052 | 0.003411 |
|  | ENSG00000144476 | ACKR3 | 49.98 | 2.337 | 5.053 | 0.049612 |
|  | ENSG00000145014 | TMEM44 | 76.80 | 2.381 | 5.209 | 0.02279 |
|  | ENSG00000187957 | DNER | 1096.11 | 2.386 | 5.228 | 8.24E-14 |
|  | ENSG00000100767 | PAPLN | 589.80 | 2.387 | 5.229 | 1.01E-04 |
|  | ENSG00000182885 | ADGRG3 | 188.41 | 2.429 | 5.385 | 1.41E-05 |
|  | ENSG00000168758 | SEMA4C | 1813.02 | 2.430 | 5.390 | 4.47E-11 |
|  | ENSG00000125810 | CD93 | 7247.91 | 2.441 | 5.430 | 1.88E-09 |
|  | ENSG00000135636 | DYSF | 339.49 | 2.461 | 5.505 | 6.40E-05 |
|  | ENSG00000197632 | SERPINB2 | 17290.89 | 2.497 | 5.644 | 5.77E-09 |
|  | ENSG00000116661 | FBXO2 | 113.99 | 2.507 | 5.684 | 1.27E-04 |
|  | ENSG00000020633 | RUNX3 | 5934.44 | 2.524 | 5.753 | 2.05E-16 |
|  | ENSG00000124875 | CXCL6 | 599.56 | 2.534 | 5.793 | 4.09E-05 |
|  | ENSG00000107249 | GLIS3 | 2069.56 | 2.560 | 5.899 | 1.61E-08 |
|  | ENSG00000173404 | INSM1 | 55.07 | 2.590 | 6.019 | 0.016021 |
|  | ENSG00000178726 | THBD | 1279.17 | 2.595 | 6.041 | 5.63E-21 |
|  | ENSG00000121743 | GJA3 | 237.49 | 2.659 | 6.316 | 3.02E-08 |
|  | ENSG00000108342 | CSF3 | 747.58 | 2.666 | 6.345 | 1.27E-06 |
|  | ENSG00000198682 | PAPSS2 | 671.09 | 2.689 | 6.447 | 2.28E-09 |
|  | ENSG00000103888 | CEMIP | 126.42 | 2.720 | 6.591 | 0.03232 |
|  | ENSG00000176490 | DIRAS1 | 31.42 | 2.724 | 6.609 | 0.044149 |
|  | ENSG00000137801 | THBS1 | 49354.93 | 2.726 | 6.617 | 8.07E-14 |
|  | ENSG00000115594 | IL1R1 | 4886.01 | 2.752 | 6.735 | 1.26E-14 |
|  | ENSG00000153132 | CLGN | 78.23 | 2.781 | 6.871 | 9.17E-04 |
|  | ENSG00000133116 | KL | 83.75 | 2.926 | 7.601 | 1.74E-05 |
|  | ENSG00000169908 | TM4SF1 | 123.69 | 2.941 | 7.678 | 8.08E-05 |
|  | ENSG00000105509 | HAS1 | 1193.27 | 2.980 | 7.887 | 2.22E-07 |
|  | ENSG00000128342 | LIF | 298.30 | 3.068 | 8.384 | 7.63E-10 |
|  | ENSG00000165105 | RASEF | 32.71 | 3.122 | 8.708 | 0.004528 |
|  | ENSG00000131459 | GFPT2 | 73.26 | 3.187 | 9.107 | 2.34E-06 |
|  | ENSG00000169252 | ADRB2 | 156.46 | 3.317 | 9.968 | 2.80E-11 |
|  | ENSG00000168874 | ATOH8 | 173.47 | 3.413 | 10.650 | 6.75E-12 |
|  | ENSG00000003436 | TFPI | 266.19 | 3.447 | 10.903 | 1.09E-12 |
|  | ENSG00000047936 | ROS1 | 39.52 | 3.453 | 10.947 | 2.20E-04 |
|  | ENSG00000164647 | STEAP1 | 59.00 | 3.459 | 10.994 | 2.99E-06 |
|  | ENSG00000276107 | THBS1-IT1 | 30.24 | 3.467 | 11.055 | 0.001579 |
|  | ENSG00000145623 | OSMR | 44.94 | 3.475 | 11.122 | 3.03E-05 |
|  | ENSG00000136379 | ABHD17C | 186.03 | 3.556 | 11.761 | 1.47E-13 |
|  | ENSG00000100234 | TIMP3 | 77.14 | 3.581 | 11.968 | 4.34E-06 |
|  | ENSG00000228382 | ITPKB-IT1 | 22.15 | 3.625 | 12.339 | 0.025535 |
|  | ENSG00000138685 | FGF2 | 197.16 | 3.632 | 12.395 | 3.52E-14 |
|  | ENSG00000215196 | BASP1-AS1 | 19.11 | 3.653 | 12.578 | 0.012887 |
|  | ENSG00000198796 | ALPK2 | 20.57 | 3.709 | 13.076 | 0.012396 |
|  | ENSG00000143434 | SEMA6C | 41.16 | 3.745 | 13.408 | 2.89E-05 |
|  | ENSG00000146072 | TNFRSF21 | 31.32 | 3.802 | 13.944 | 0.001133 |
|  | ENSG00000289724 | ENSG00000289724 | 24.58 | 3.833 | 14.248 | 0.048066 |
|  | ENSG00000153162 | BMP6 | 929.01 | 4.153 | 17.793 | 3.35E-36 |
|  | ENSG00000120875 | DUSP4 | 2365.38 | 4.196 | 18.322 | 3.16E-85 |
|  | ENSG00000001617 | SEMA3F | 21.32 | 4.380 | 20.817 | 0.001856 |
|  | ENSG00000175003 | SLC22A1 | 431.29 | 4.971 | 31.362 | 5.02E-29 |
|  | ENSG00000162892 | IL24 | 6917.19 | 5.125 | 34.897 | 1.07E-44 |
|  | ENSG00000105792 | CFAP69 | 10.68 | 5.257 | 38.233 | 0.020384 |
|  | ENSG00000258926 | ENSG00000258926 | 211.99 | 5.648 | 50.143 | 2.65E-32 |
|  | ENSG00000183785 | TUBA8 | 8.19 | 6.471 | 88.701 | 0.010268 |
|  | ENSG00000287535 | ENSG00000287535 | 18.17 | 6.651 | 100.488 | 0.001133 |
|  | ENSG00000274944 | ENSG00000274944 | 18.83 | 7.679 | 204.965 | 3.88E-05 |
