# Supplementary material for: Bordetella adenylate cyclase toxin elicits chromatin remodeling and transcriptional reprogramming that blocks differentiation of monocytes into macrophages
Source: mBio. 2025 Mar 19;16(4):e00138-25. doi: 10.1128/mbio.00138-25 (PMC11980580; doi:10.1128/mbio.00138-25)
Supplement: Table S2 — Primers used in this study. [file mbio.00138-25-s0005.docx]

**Supplementary Table S2**

**The list of primers used in this study**

| **Gene symbol** | **Forward primer** | **Reverse primer** | **Amplicon size** |
| --- | --- | --- | --- |
| FCGR2B | ACCATCACTGTCCAAGCTC | CCTGGGAGAGCTGAAATCC | 139 |
| SIGLEC1 | CAGTAACAGAGGAGCCCAG | AGCAGTTGAAGTCCACCTC | 80 |
| SPP1 | CCATACCAGTTAAACAGGCTG | TCAGGGTTTAGCCATGTGG | 93 |
| FBP1 | GATGTTCATCGCACTCTGG | CAGTCTCAGCTTTCCATTGG | 80 |
| CCL8 | AAGCTGTGATCTTCAAGACC | GCTTCATGGAATCCCTGAC | 80 |
| CXCL10 | ACGTGTTGAGATCATTGCT | AAATTCTTGATGGCCTTCGA | 80 |
| NCF1 | GTTCCTGGTGAAATGGCAG | TTATGGAACTCGTAGATCTCGG | 74 |
| TNIK | GAAGTTAAACTAGTGGACTTTGGAG | GGGAGTTCCAATGAAAGTATTCC | 80 |
| CCL7 | CTTGCTCAGCCAGAAAGAC | GCAGGTAGTTGAAGTATTAATCCC | 91 |
| CCR5 | ATCTTCTTCATCATCCTCCTGAC | CAAACACAGCATGGACGAC | 60 |
| PFKFB3 | AAGAGTGCAGAGGAGATGC | TAGATGGATTCCACACGGC | 94 |
| VEGFA | CAGAATCATCACGAAGTGGTG | GAAGATGTCCACCAGGGTC | 83 |
| DLL1 | GTATCCGCTATCCAGGCTG | TAGTTCAGGTCCTGGTTGC | 102 |
| SLC39A14 | ACTCAACCACCTGGATGTG | ACTAAAGCACGTGGAGAGG | 84 |
| CD93 | AGAAGAAGGAGAAGAAGCCC | CAGGTGTCGGACTGTACTG | 101 |
| RUNX3 | ATGACGAGAACTACTCCGC | AGGGTGAAACTCTTCCCTC | 117 |
| IL1R1 | GTGGTATAAGGATTGCAAACCTC | ACATTCATCACGATGAGCCT | 80 |
| DUSP4 | TACAAGTGCATCCCAGTGG | TCACGGCATCGATGTACTC | 84 |
| IL24 | TTACAGGACCAGAGCAAGC | TGGACAAGGTAACAGCTCTC | 102 |
| EGR2 | GACCATCTTTCCCAATGCC | TCAATGTTGATCATGCCATCTC | 80 |
| FCN1 | AATCATTCAAGGTGGCTGAC | GTTAGAGAATTACCCGCACTG | 84 |
| VSIG4 | TCTCCTTGTGCTGTATGGT | CGTAGACATGCTCTTGTTGG | 80 |
| CSF1 | AACAGTTGAAAGATCCAGTGTG | TATCTCTGAAGCGCATGGT | 86 |
| FPR3 | AACTCAGCTAGTGGTGTGG | AGATCCACAGAACGGTGTG | 104 |
| STAT1 | GATTTAATCAGGCTCAGTCGG | TTCTGACTTTACTGTCAAGCTC | 80 |
| SLC1A3 | CTGTCATTGTGGGTACAATCC | GAAAGGAGAAGTACTTGACTTCC | 80 |
| SLCO2B1 | ACATCAAGCTGTTCGTTCTG | AGATGGAGCTCTTTAGGTAGC | 80 |
| GBP1 | ACTGAGAAGATGGAGAACGA | CTCCTGTTCCTGAAGTTTAAGAG | 80 |
| HLA.DRA | ACTGAGGACGTTTACGACTG | TGGAGCATCAAACTCCCAG | 80 |
| HLA.DMB | TCTCCCATTTAGCCTTAACCC | GGAAATGTGCCATCCTTCTG | 142 |
| CD74 | AGAGGACCATGTGATGCAC | AGCTCTCAAAGACCTTCCAG | 136 |
| STEAP4 | ACTTGCGAGTTGTTATGGAG | TCAATCCCAGTGATCTTCCA | 122 |
| HLA.DPA1 | CCTGTGAAATACTGTAAAGGTGAC | AGATGGAGTTTGTAGGGCAG | 88 |
| SERPINB2 | CAGAACCTCTTCCTCTCCC | TAAACTGAAGCACCTTGGC | 105 |
| RASAL1 | AAGGTACGCCTGATTGAGG | CTAGCAGTGTCCTCCTCTG | 106 |
| GBP5 | CTGTTCCTACATCTTTAGCCA | AGGTTCTTTAGACGAGATCCA | 80 |
| NCF1C | CAGCACTATGTACATGTTCCTG | TCGTAGATCTCGGTGAAGC | 80 |
| MRC1 | GTGGCACCAGGCGAGGAAAAG | CGGTCACTCCACTGCCAACC | 168 |
| CD36 | GCTGTCATTGGTGCTGTCCTGG | GCTGCTGTTCATCATCACTTCCTGTGG | 204 |
| CD11b | GGGAAGTGGCAAGGAATGTA | CTGCGTGTGCTGTTCTTTGT | 214 |
