# Supplementary material for: Bordetella adenylate cyclase toxin elicits chromatin remodeling and transcriptional reprogramming that blocks differentiation of monocytes into macrophages
Source: mBio. 2025 Mar 19;16(4):e00138-25. doi: 10.1128/mbio.00138-25 (PMC11980580; doi:10.1128/mbio.00138-25)
Supplement: Table S3 — Primers used for ChIP-qPCR. [file mbio.00138-25-s0006.docx]

**Supplementary Table S3**

**The list of primers used for ChIP-qPCR**

| Gene symbol | Forward primer | Reverse primer | Amplicon size (bp) |
| --- | --- | --- | --- |
| EGR2 | AGGTGAGCGGCTGAAGAC | AGCGGCCCTAGCTTAGTGT | 98 |
| SERPINB2 | ATTGACGCTCTGCTGCTCTT | GTGCTCAAGGCATCAACTGC | 86 |
| HLA-DRA | GCCACAGTGTGTTCAAAGCC | CATGCAGCCGTTCTCACAAG | 90 |
| JUN | CCTGGCTCACAGTAGGCTTT | TTTCTTCACTCCGGGCACTC | 73 |
| CD74 | CCAGGGACAGAGACCAGAGA | GGGAGGATGGGACTGTGTTG | 74 |
| FCGR2B | TGGCATTCAACCCGAAACCT | TCCCTGTTCTCACTGCAACG | 75 |
| MRC1 | TGGAAACTGAAGGGGATGAGC | GTCCCTCAGCAGACAACCTC | 110 |
| CSF1 | TAAAGAACTCCAGTGCGGGG | TGAGGCTGGCATTTGGACTT | 102 |
| VSIG4 | TCACCACCAGCTGGAACCT | TTGGGTCTTAGCCTTGCTCTTT | 110 |
| FCN1 | GCAGCCCTTGTGAATGTTGG | TACGGCCTGCCAAAGGTATG | 98 |
| GBP1 | TAAGTGCCAGGGGTTAGGTG | CTGCCTATTCTTTGAGAGGTGC | 80 |
| FCGR1A | GCTGAGGCTTCTCTCGTCTC | AGCAAGAGCTGACTGACACA | 88 |
| PSMB9 | TTACCCCCTAACTGCATCACC | AGGGGATAGGGGAAGTGGAA | 76 |
